# Relationship of ventral striatum activation during effort discounting to clinical amotivation severity in schizophrenia

**DOI:** 10.1038/s41537-021-00178-9

**Published:** 2021-10-08

**Authors:** Greer E. Prettyman, Joseph W. Kable, Paige Didier, Sheila Shankar, Theodore D. Satterthwaite, Christos Davatzikos, Warren B. Bilker, Mark A. Elliott, Kosha Ruparel, Daniel H. Wolf

**Affiliations:** 1 Department of Psychiatry, University of Pennsylvania, Philadelphia, PA 19104 USA; 2 Department of Psychology, University of Pennsylvania, Philadelphia, PA 19104 USA; 3 Center for Biomedical Image Computing and Analytics, University of Pennsylvania, Philadelphia, PA 19104 USA; 4 Penn Lifespan Informatics and Neuroimaging Center, University of Pennsylvania, Philadelphia, PA 19104 USA; 5 Department of Radiology, University of Pennsylvania, Philadelphia, PA 19104 USA; 6 Department of Biostatistics, Epidemiology & Informatics, University of Pennsylvania, Philadelphia, PA 19104 USA

**Keywords:** Biomarkers, Neural circuits

## Abstract

Motivational deficits play a central role in disability due to negative symptoms of schizophrenia (SZ), but limited pathophysiological understanding impedes critically needed therapeutic development. We applied an fMRI Effort Discounting Task (EDT) that quantifies motivation using a neuroeconomic decision-making approach, capturing the degree to which effort requirements produce reductions in the subjective value (SV) of monetary reward. An analyzed sample of 21 individuals with SZ and 23 group-matched controls performed the EDT during fMRI. We hypothesized that ventral striatum (VS) as well as extended brain motivation circuitry would encode SV, integrating reward and effort costs. We also hypothesized that VS hypoactivation during EDT decisions would demonstrate a dimensional relationship with clinical amotivation severity, reflecting greater suppression by effort costs. As hypothesized, VS as well as a broader cortico-limbic network were activated during the EDT and this activation correlated positively with SV. In SZ, activation to task decisions was reduced selectively in VS. Greater VS reductions correlated with more severe clinical amotivation in SZ and across all participants. However, these diagnosis and amotivation effects could not be explained by the response to parametric variation in reward, effort, or model-based SV. Our findings demonstrate that VS hypofunction in schizophrenia is manifested during effort-based decisions and reflects dimensional motivation impairment. Dysfunction of VS impacting effort-based decision-making can provide a target for biomarker development to guide novel efforts to assess and treat disabling amotivation.

## Introduction

Amotivation is a prominent negative symptom of schizophrenia (SZ) and a major driver of long-term disability^1–4^. While currently available antipsychotics target positive symptoms, negative symptoms including amotivation constitute a major unmet therapeutic need^5^. Despite this crucial importance, the pathophysiology of amotivation in SZ is not well understood, posing a critical barrier to therapeutic development.

Neuroeconomic approaches offer a framework to study specific aspects of motivated behavior such as effort-based decision-making (EBDM). EBDM tasks leverage reward/effort trade-off decisions as a measure of motivation^6–9^. As the amount of effort required to obtain a reward increases, the subjective value (SV) of that reward is reduced, a phenomenon known as effort discounting. Neuroeconomic techniques can model specific decision-making patterns of individuals, including those with impaired motivation.

SZ is associated with abnormal EBDM behavior, particularly reduced willingness to increase effort to obtain rewards of greater value or probability, suggesting greater subjective cost of effort^1,8,10–12^. These categorical diagnostic effects may reflect dimensional variation, as several studies in SZ show an inverse correlation between negative symptom severity and willingness to exert effort^10,13–17^. Different EBDM paradigms differ in sensitivity to categorical vs. dimensional effects^18,19^, and the measures most sensitive to the full dimensional spectrum of motivational variation may be less sensitive to categorical diagnostic discrimination.

Studies in animal models have identified a core motivation circuit comprised of dopamine projections from the midbrain ventral tegmental area (VTA) to the nucleus accumbens (NAc) within the ventral striatum (VS), and to medial prefrontal areas including ventromedial prefrontal cortex (vmPFC) and dorsal anterior cingulate cortex (dACC)^20–27^. Inhibiting dopamine signaling in the NAc reduces effort exertion^28–30^, while enhancing dopamine activity increases it^31,32^. The same circuitry has been tied to motivation in the human brain. Functional magnetic resonance imaging (fMRI) studies have implicated regions including VS, vmPFC, and dACC in EBDM^33–42^, and a PET imaging study linked dopamine function in the striatum and vmPFC to willingness to exert effort^43^. VS activation is reduced at time of reward following higher-effort performance and at time of choice for higher efforts, providing evidence for a representation of effort discounting in VS^33,35,38,40,44–46^. However, some EBDM fMRI studies did not report VS activation^36,41,47–50^, and the specific EBDM components evoking activation in different regions remains uncertain.

Despite extensive behavioral evidence of reduced willingness to exert effort in SZ, the fMRI correlates of these EBDM abnormalities have not yet been well characterized. The three such studies to date have linked SZ to abnormal activation in the VS during effort tasks^51–53^. However, these studies used very different effort constructs, and none of them used full parametric variation of both reward and effort to examine their separate and integrated (model-based SV) effects. Furthermore, these studies differed in terms of whether fMRI abnormalities were robustly captured as categorical effects of diagnosis or dimensional correlates of amotivation. Our own prior study identified dimensional but not categorical VS hypofunction in SZ^8^; however, the correlation was only significant for out-of-scanner effort behavior and not clinical amotivation, and the fMRI reward task did not involve effort decisions. Thus, further work is needed to clarify which components of EBDM relate to VS hypofunction and whether these are best understood as categorical or dimensional.

**Fig. 1:**
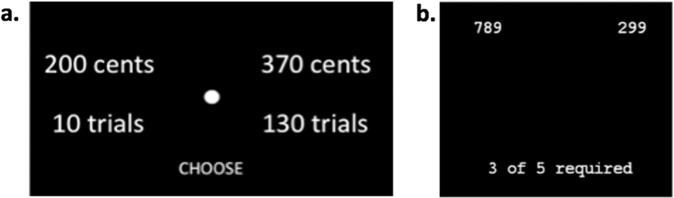
Example trial from fMRI EDT Task. Participants selected between a low effort/low money (EASY) trial and a parametrically varied higher-effort/higher-reward (HARD) option (**a**). The effort “trials” participants were asked to consider were repetitions of a “Bigger Number Task” (BNT) utilized in previous motivation tasks and here performed out of scanner (**b**).

The current study sought to address the above gaps in current understanding by investigating EBDM in patients with SZ and healthy controls using an fMRI effort discounting task (EDT, Fig. 1) that independently manipulated reward and effort levels across a wide range. This allowed us to analyze activation during decision-making as well as activation that specifically relates to effort cost, reward, and subjective value. We hypothesized that VS and a broader motivation network encode SV, with parametrically-modeled reward and effort exerting opposite effects. Based on prior literature linking amotivation in SZ specifically to VS hypofunction, we hypothesized that the SZ group would exhibit lower activation selectively within VS during effort-based decisions, and that this average effect of diagnosis would reflect dimensional severity of clinical amotivation. Finally, we expected that application of neuroeconomic modeling would increase sensitivity and interpretability of clinical effects. Specifically, we predicted that amotivation would reflect greater suppression of VS activation by effort cost.

## Results

### Behavioral results

Relative to controls, SZ showed elevated clinical amotivation and other symptoms, as expected (Table 1). The primary measure of behavioral motivation, logB, did not differ between groups (CT: −0.31 ± 0.59; SZ: −0.41 ± 1.05; *t* = 0.38; *p* = 0.70) nor was it significantly correlated with clinical amotivation across all participants (*r* = 0.04; *p* = 0.81) or in SZ patients (*r* = 0.03; *p* = 0.89). As expected, the a priori linear model fit behavioral data better than the hyperbolic or parabolic models; model fit did not show significant group differences or correlations with amotivation (see Supplementary Discussion).

**Table 1:** Demographic and clinical information for patient and control groups.

Variable	Control (*n* = 23)	Schizophrenia (*n* = 21)	*p* value
Gender (% female)	43% (10F/13M)^a^	43% (9F/12M)	0.99^b,c^
Handedness (% right)	96% (22R/1L)	95% (19R/2L)	0.59
Smoke (% yes)	9% (2Y/21N)	24% (5Y/16N)	0.23
Age (yrs)	32.2 (10.3, 19–54)^d^	36.6 (7.7, 22–54)	0.13^e^
Education (yrs)	14.7 (2.0, 12–18)	13.7 (1.5, 12–18)	0.07
Parental education (yrs)	14.5 (2.1, 12–19)	13.1 (2.7, 9–19)	0.06
CAINS amotivation	0.61 (0.56, 0–1.9)	1.56 (0.81, 0.4–2.8)	<0.001
CAINS total avg.^f^	0.69 (0.45, 0.1–2.0)	1.41 (0.57, 0.4–2.5)	<0.001
Global SANS avg.^g^	0.20 (0.41, 0–1.5)	2.15 (0.89, 0.5–3.5)	<0.001
Global SAPS avg.	0.00 (0.11, 0–0.5)	1.11 (1.13, 0–4.3)	<0.001
Depression symptoms^h^	0.13 (0.34, 0–1.0)	3.43 (3.83, 0–12.0)	<0.001
Cognitive performance^i^	0.45 (0.36, −0.32–0.95)	−0.30 (0.71, −1.7–0.63)	<0.001
Antipsychotic dose^j^	NA	421 (270, 0–1226)	NA

^a^Categorial variables are reported as percentage (proportion).

^b^All *p*-values in the table are two-tailed, uncorrected.

^c^Fisher’s exact test was used to compare proportions for categorical variables.

^d^Continuous variables are reported as mean (SD, min–max).

^e^Student’s *t* test used for comparing group means.

^f^CAINS scores are averages across items. See details in Supplementary Materials.

^g^SANS and SAPS scores reflect the averages across items.

^h^Calgary Depression Scale for Schizophrenia.

^i^Calculated from Penn CNB as *z*-scores across all subjects from both groups.

^j^Antipsychotic dose in chlorpromazine equivalents; antipsychotic treatment: none (*n* = 1), 1st gen (*n* = 3), 2nd gen (*n* = 17).

### Neuroimaging results

#### Task activation

Across the full sample, the non-parametric task regressor (mean response across trials) revealed activation in brain regions involved in valuation and decision-making. Specifically, the VS ROI was strongly activated (*p* < 0.001; peak *t* = 7.19; MNI −20,12,0); however, a much smaller region (78 voxels vs. 509 voxels) of anteroventromedial VS bordering vmPFC was task-deactivated (*p* < 0.001; peak *t* = −7.00; −4,16,−8). dACC was activated by task (*p* < 0.001; peak *t* = 8.82; 2,26,36) while vmPFC was deactivated (*p* < 0.001; peak *t* = −8.52; 2,46,−14). An exploratory whole-brain analysis showed these effects and revealed additional task-modulated regions (see Supplementary Figs. 2 and 3).

**Fig. 2:**
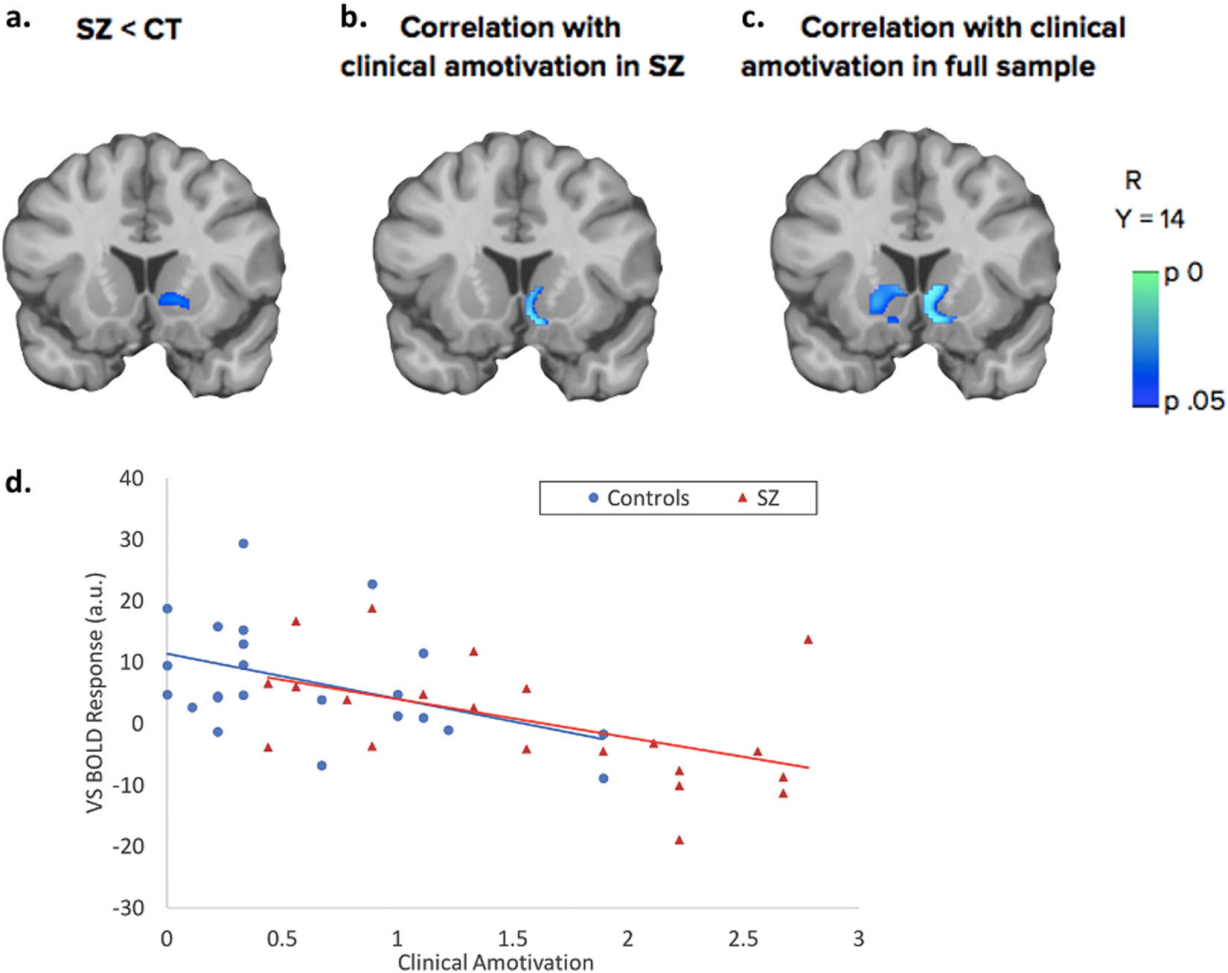
Clinical effects on VS activation to effort-based decisions. Individuals with schizophrenia (SZ) had reduced VS activation during task decisions compared with control participants (CT) (**a**). In patients with SZ, severity of clinical amotivation correlated inversely with VS activation during task decisions (**b**). A negative correlation between VS activation to task and clinical amotivation was also observed across all participants, controlling for group (**c**). In **a**–**c**, significance is determined within VS ROI. Descriptive scatterplot (contrast parameters extracted from entire VS ROI) shows this dimensional relationship across the full sample, separately labeling SZ in red and CT in blue (**d**).

As hypothesized, SZ exhibited significantly less VS activation to task compared to CT (*p* = 0.027; peak *t* = 3.38; 12,12,−4; Fig. 2a, see Supplementary Fig. 4 for selectivity of VS effect). Furthermore, in SZ greater clinical amotivation was associated with reduced task activation in VS (*p* = 0.004; peak *t* = −4.92; 8,14,−14; Fig. 2b). In controls, VS activation also inversely correlated with amotivation at a trend level (*p* = 0.073). When group and amotivation were jointly modeled across all participants, the VS dimensional effect was still significant (*p* = 0.028; peak *t* = −4.95; 6,14,0; Fig. 2c, d) but the group effect was no longer significant. The categorical and dimensional VS results were not driven by other negative symptoms or by other potential confounds (see Supplementary Tables 2 and 3). Neither dACC nor vmPFC activation showed group effects or amotivation correlations in any of these analyses (*p*’s > 0.1); In whole-brain analyses the dimensional effect across all participants was most significant in VS (*p* = 0.007; whole-brain peak *t* = −5.23; 6,16,2; see Supplementary Fig. 5). The dimensional relationship in dorsal striatum was also significant, unlike the categorical effect (see Supplementary Figs. 4 and 5 and Supplementary Discussion).

#### Effort and reward

**Fig. 3:**
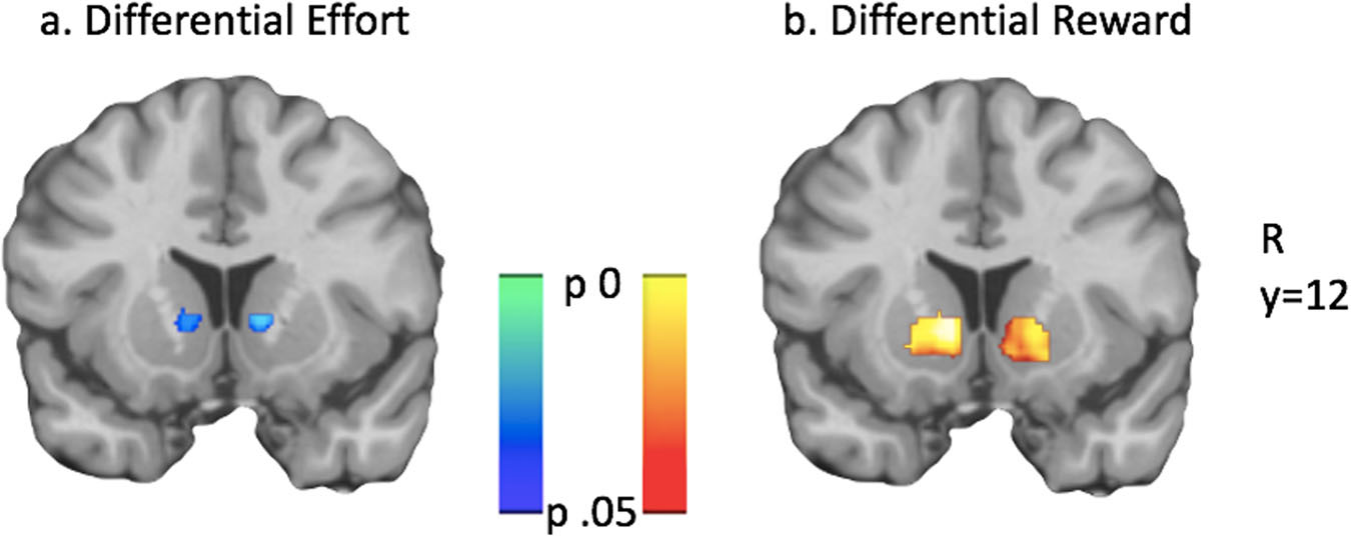
Effort and reward exert opposite effects in VS. Differential effort (HARD–EASY) suppressed VS activation (**a**), while differential reward positively activated VS (**b**). Significance is determined within VS ROI.

Parametric models were used to further parse these average task results and identify effects related to across-trial variation in effort, reward, and integration as SV. Across all participants, reward and effort exerted opposite effects in the directions expected for valuation regions. Differential effort (HARD–EASY) deactivated VS (*p* = 0.01; peak *t* = −4.09; 10,14,2; Fig. 3a). Differential effort even more robustly deactivated dACC (*p* < 0.001; peak *t* = −4.39; 4,34,28), with no effect in vmPFC. Whole-brain analysis also identified VS and dACC de-activation, as well as other deactivated areas including bilateral insula, middle frontal gyrus, and occipital cortex; no areas were positively correlated with effort. Conversely, differential reward positively correlated with VS activation (*p* < 0.001; peak *t* = 4.95; 10,4,0; Fig. 3b), but did not activate vmPFC or dACC. Whole-brain analysis showed the VS differential reward effect as well as positive activation in task-active regions (*p* < 0.01).

We found no significant group differences in activation related to differential effort costs or reward values in VS, secondary ROIs, or whole-brain analysis. Greater CAINS amotivation in SZ was associated with increased vmPFC activation to effort costs (*p* = 0.022; peak *t* = 3.21; −2,46,−18). VS and dACC showed no differential effort correlation in SZ, and none of the three regions showed effort correlations across the full sample (*p*’s > 0.1). Whole-brain analyses did not identify any significant regions in these effort correlation analyses. There were no correlations between amotivation and differential reward magnitude in VS, secondary ROIs, or whole-brain analysis, within the SZ group or across the full sample (*p*’s > 0.1).

#### Subjective value

Two parametric models for individualized subjective value (SV) were compared; both have been employed in prior discounting literature but rarely in the same study^54^. The first modeled differential SV between the two presented options. The second SV model took into account only the SV of the chosen option.

**Fig. 4:**
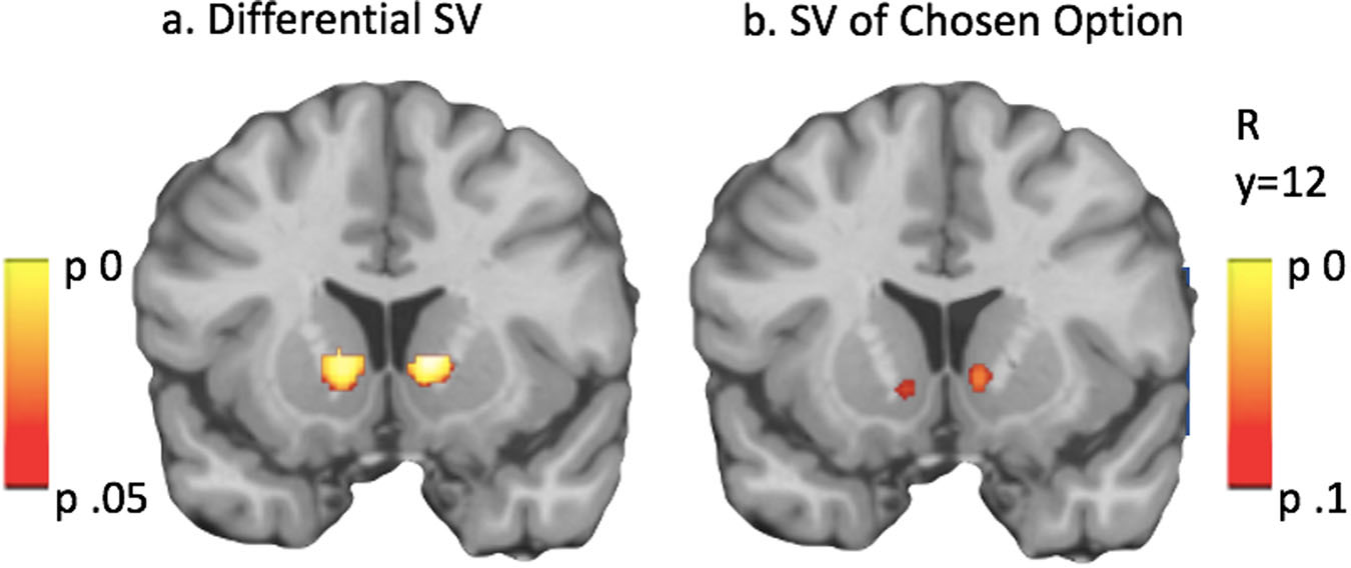
Comparison of two models for subjective value. Bilateral VS was activated by both differential subjective value (SV, HARD–EASY) (**a**), and by SV of the chosen option (**b**). Significance is determined within VS ROI. Display threshold is set below significance levels to illustrate bilaterality of the chosen value effect (significant on right, trend on left).

Bilateral VS was associated with differential SV in the full sample (*p* = 0.006; peak *t* = 5.34; 12,10,2, Fig. 4a). dACC also strongly activated to differential SV (*p* < 0.001; peak *t* = 5.20; 4,30,34), while vmPFC showed no effect. Whole-brain analysis showed the effects in VS and dACC, and revealed additional differential SV activation in frontal cortex, occipital cortex, parietal cortex, insula, and thalamus.

SV of the chosen option also parametrically activated VS (*p* = 0.048; peak *t* = 3.33; 10,14,−2, Fig. 4b). Although differential SV showed stronger VS activation than chosen SV, chosen SV activation was more spatially selective for VS than differential SV, and centered in more antero-ventral regions tied to reward as opposed to non-valenced/salience effects^55^ (see Supplementary Fig. 6). Chosen SV did not show any effects in dACC or vmPFC or in whole-brain analysis.

Group analysis of differential SV revealed a group difference only in vmPFC, with SZ showing greater activation than CT (*p* = 0.01; peak *t* = 3.80; 4,38,−4). VS and dACC did not show any group differences or association of differential SV with clinical amotivation in SZ or across all participants. No significant group differences or associations between amotivation and chosen SV were found in VS, dACC, vmPFC, or whole-brain analysis. In addition, no correlations were found across the whole brain in any region.

The above imaging findings are summarized in Table 2.

**Table 2:** Summary of findings for each contrast tested in the primary VS ROI and secondary (dACC, vmPFC) ROIs.

	VS	dACC	vmPFC
*Model 1 Task*			
Average response	Activated	Activated	Deactivated
Group difference	SZ < CT	ns^a^	ns
Correlation with amotivation SZ	Negative	ns	ns
Correlation with amotivation transdiagnostic	Negative	ns	ns
*Model 2 Effort*			
Average response	Deactivated	Deactivated	ns
Group difference	ns	ns	ns
Correlation with amotivation SZ	ns	ns	Positive
Correlation with amotivation transdiagnostic	ns	ns	ns
*Model 2 Reward*			
Average response	Activated	ns	ns
Group difference	ns	ns	ns
Correlation with amotivation SZ	ns	ns	ns
Correlation with amotivation transdiagnostic	ns	ns	ns
*Model 3 Differential SV*			
Average response	Activated	Activated	ns
Group difference	ns	ns	SZ > CT
Correlation with amotivation SZ	ns	ns	ns
Correlation with amotivation transdiagnostic	ns	ns	ns
*Model 4 Chosen SV*			
Average response	Activated	ns	ns
Group difference	ns	ns	ns
Correlation with amotivation SZ	ns	ns	ns
Correlation with amotivation transdiagnostic	ns	ns	ns

^a^ns = not significant at *p* < 0.05 threshold.

## Discussion

We applied an effort discounting task to examine normal and abnormal motivation processes. We found that ventral striatum, the core brain region underpinning motivation, was hypoactive during effort-based decision-making in schizophrenia and this hypofunction correlated with clinical amotivation severity. Across the full sample, VS activation corresponded to model-based subjective value, integrating reward magnitude and effort costs; however, the key clinical findings in VS were not captured by model-based analyses.

There are only three published studies examining fMRI effort discounting in schizophrenia^51–53^; our results are broadly consistent in identifying abnormal VS function. However, there are key differences in both methods and results. Huang et al.^51^ used the EEfRT^6^, a task which manipulates reward magnitude (and probability) but does not vary effort levels associated with the hard choice. As we did, they found reduced VS activation in SZ during the choice phase; in contrast, they observed abnormal lack of VS modulation by reward, which we did not find. Culbreth et al.^53^ applied an effort paradigm previously used to examine valuation vs. decision-difficulty responses^56^. They also found the same inverse correlation of VS activity and clinical amotivation we observe, however, it is unclear whether the decisional-difficulty contrast they examined captures reward-effort trade-offs. Park et al.^52^ adapted a paradigm which manipulated reward and effort cues without choices^35^. They did not find abnormalities in SZ during the valuation phase, but instead found lower VS activation correlating with amotivation in SZ during effort performance, and heightened VS activation in SZ during the feedback phase.

A more extensive literature using non-effort tasks links VS hypofunction to negative symptoms and amotivation^57–62^. Our own prior work showed a correlation in SZ between behavioral motivation (measured with a progressive ratio variant of our EDT) and VS activation in a non-effort reward task^8^. The present study demonstrates that VS hypofunction is present during reward-effort trade-off decisions and that this explicitly motivational decision process correlates with amotivation measured clinically. However, the finding of VS hypofunction across tasks with and without explicit effort or motivation components raises important questions regarding psychological specificity. Striatal dysfunction has been linked to deficits in reward anticipation, reinforcement learning, and prediction error signaling^63^, all of which may relate to VS hypofunction. One possibility is that amotivation in SZ is associated with primary VS hypofunction that manifests in any fMRI measure of VS activation (“pseudospecificity”). An alternative and compatible possibility is that reward and non-reward tasks^64,65^ may probe the motivational function of VS even when motivation is not an explicit task focus. Our findings strengthen the connection between VS hypofunction, motivated decisions, and clinical amotivation, while also pointing to the need for work directly comparing related constructs.

Notably, we confirmed the primary dimensional relationship between VS hypofunction and amotivation, consistent with the RDoC framework of psychopathology^66^. The literature supports both categorical and dimensional VS hypofunction, but this varies across studies including across the three prior effort fMRI studies. Only the categorical effect was robust in Huang et al.; Culbreth et al. found only robust dimensional effects; and Park et al. reported a dimensional hypofunction effect but an opposite-direction categorical effect (in a different contrast)^51–53^. Our results support the view that motivation impairment is *not* a characteristic of schizophrenia in general, instead manifesting only in a subset, to varying degrees. A primary dimensional effect will often produce a secondary categorical (average) effect, but can also reduce apparent categorical effects through increased intra-group variability. Detection of expected dimensional amotivation effects can be facilitated by measures that capture a wide range of motivation with limited sensitivity to other common features including psychomotor slowing, cognitive impairment, and positive symptoms. Some prior work (e.g., Reddy et al.^19^) recommends using categorical discrimination sensitivity as a criterion in evaluating which motivation paradigms should be used in clinical trials. We disagree, and argue that understanding the pathophysiology of amotivation and developing biomarkers or treatments for this symptom requires prioritizing dimensional over categorical sensitivity, in line with an understanding that amotivation reflects a dimensional neurobehavioral construct.

Our whole-brain analyses of task activation demonstrated that group differences and amotivation correlations were especially robust in VS, suggesting a dominant contribution of this region to amotivation in SZ. While this relative VS-selectivity is broadly consistent with the prior work in SZ noted above, it is surprising considering extensive human and animal research identifying a broader circuit in EBDM and value-based decisions, in particular dACC and vmPFC^55,67–69^. Unusual VS sensitivity of our task contrast or analysis cannot explain our findings, as other more strongly task-activated regions did not exhibit the categorical or dimensional clinical effects. Culbreth et al. also identified VS-selective amotivation correlations with a contrast that activated other regions more robustly, and SZ abnormalities in Park et al. also appeared selective for VS^52,53^. However, Huang et al. found similar reductions in VS, posterior cingulate and vmPFC^51^. We argue that while many regions are involved in normal motivation, the selectivity of VS for this psychological process and selectivity of VS dysfunction in amotivated individuals with SZ contribute to its preferential identification in fMRI studies such as ours.

Across all participants, our parametric reward and effort manipulations elicited hypothesized opposite-direction effects in valuation regions (positive reward and negative effort response). Consistent with encoding of integrated subjective value (reward discounted by effort), participant-specific SV models revealed activation in VS and other valuation regions. Compared to differential SV, chosen SV elicited activation that was more selective for VS, as well as more centered in ventromedial (NAc) positive-valence vs. valence-insensitive salience regions of VS^55^. This suggests that NAc selectively encodes chosen value in our paradigm. The more spatially extended differential SV response may also reflect covarying task-evoked psychological processes, consistent with reports that dACC and other task-active regions respond to decision difficulty (which is greater when the difference in SV between hard and easy options is reduced)^70,71^. Contrary to expectations, vmPFC activation did not reflect SV, vmPFC differential SV response was higher in SZ, and vmPFC effort-related activation positively correlated with amotivation; as vmPFC is strongly deactivated by decision-difficulty this might occlude valuation signals. We note that the specific regional pattern associated with SV in prior fMRI studies in healthy volunteers varied substantially across studies. For example, while some prior effort fMRI studies in healthy volunteers reported discounted SV signals in VS^33,35,38,40,44–46^ others failed to find SV effects in VS^36,41,47–50^. While some of the latter studies concluded that VS does not encode effort-discounted SV^41,49^, our results indicate it does; across-study variation likely reflects differences in task design and modeling. An unusual feature of our paradigm is the inclusion of a high effort range, such that on many trials the SV of the harder option is strongly negative. This likely increases the impact of effort on behavior as well as altering regional sensitivity to parametric regressors; for example, the chosen SV regressor has almost exclusively positive values while the differential SV regressor has many negative values.

Despite finding expected parametric responses in VS, our hypothesized clinical findings in VS were only significant for overall activation during decisions, without any significant relationship to the parametric factors contributing to those decisions. In particular, we did not find support for our hypothesis that those with greater amotivation would show greater suppression of VS to differential effort cost.

The absence of model-based clinical correlates indicates that employing quantitative model-based paradigms may not always enhance sensitivity in relating fMRI activation to particular psychological processes. The complexity of model-based fMRI findings when examining individual differences has been highlighted by Lebreton et al.^72^, particularly with regard to effects related to adaptive coding and regressor scaling. Furthermore, compared to simple across-trial task regressors, parametric regressors may be more affected by trial-by-trial variation in noise/artifact or other psychological processes, impacting their reliability and driving unexplained individual variability. While complicating interpretation, the examination of simple task contrasts alongside different parametric models is a strength of our study, and work employing only a single approach may provide a misleading simplicity. Importantly, the fact that our clinical fMRI effects are found in the simpler task-based contrast means they are not driven by any potential mis-specification of the discounting model. Our study highlights potential limitations of parametric model-based analyses particularly when applied in smaller samples, and also emphasizes the importance of including standard task activation measures which may be more statistically robust. Future studies examining the role of VS in representing effort costs in individuals with amotivation should utilize samples large enough to accommodate individual differences in task strategy as well as potentially noisier parametric measures, should incorporate designs that disentangle SV from decision difficulty, and should compare different discounting tasks and modeling approaches within the same sample.

Contrary to expectation, we did not find a relationship between clinical amotivation and behavioral measures of motivation in the EDT. While many studies do report expected behavioral effects^1,8,10–12,51^, others do not report reduced effortful choices in schizophrenia, particularly at low values of reward^6,10,12,13,73^. This heterogeneity, and the absence of behavioral abnormalities here, may reflect the complexity of factors impacting individual differences in effort decisions (see Supplementary Discussion). Some aspects of our paradigm that were designed to increase interpretability of fMRI measures (e.g., parametric modulation of both reward and effort with random trial ordering, and deferred effort performance) could potentially make the behavioral outcome more vulnerable to cognitive confounds or variation in task strategy. Rather than only exhibiting a reduction in willingness to exert effort, some individuals with schizophrenia likely allocate effort in suboptimal ways^74,75^, and this may be exacerbated in more complex or abstract tasks. We did observe some across-subject variability in the best-fitting SV model, supporting this possibility.

Nonetheless, our task does produce expected effects of reward and effort on choice behavior in the schizophrenia group and across the full sample. Furthermore, the task produces VS activations to subjective value, reward, and effort in expected directions across the full sample, and most importantly it produces VS activation across trials that correlates with clinical amotivation in the expected direction in both SZ and across the full sample. This suggests that VS activation with fMRI may have better sensitivity to detect amotivation differences than behavioral measures, although this could vary based on the specific task and specific sample. Although the severity of clinical amotivation in our patient sample is similar to that reported in other effort discounting fMRI studies, and the range was sufficient to identify correlations with VS activation, it is possible that inclusion of more patients at the most severe end of the amotivation range would have enhanced our ability to detect behavioral differences.

In addition to the limitations in the discussion above, several other limitations deserve to be emphasized. Our group matching was imperfect and certain demographic factors show trend differences between groups, although demographic variables did not explain key results (see Supplementary Discussion). In addition, we studied primarily patients with chronic schizophrenia on antipsychotics. While this facilitates generalizability to the typical schizophrenia population, antipsychotics affect dopamine signaling and could alter VS activation^76^. As we did not expect our neurobehavioral measures to distinguish primary from secondary amotivation, we did not include a measure selective for primary negative symptoms. Amotivation severity did correlate with positive symptom severity in our patients, suggesting both primary and secondary symptoms are present; however, our findings were not explained by positive symptoms or other common contributors to secondary negative symptoms including depression or antipsychotic dosage. Given these limitations, it will be important to evaluate the reported effects in larger medication-naive populations with an even wider range of amotivation severity.

These limitations notwithstanding, our results provide important new evidence regarding normal and abnormal patterns of activation during effort discounting. In particular, our findings provide strong support for a dimensional relationship between VS hypofunction during effort-based decision-making and clinical amotivation severity. A better understanding of specific impairments in effort-based decision-making will ultimately lead to better strategies for treating disabling amotivation. As clinical amotivation and reduced willingness to exert effort in behavioral tasks are also observed in first episode psychosis and clinical high risk for psychosis^77–79^ neurobehavioral biomarkers of amotivation may also provide targets for early detection and intervention.

## Methods

### Participants

Of 50 enrolled participants, 44 had analyzable data, including individuals with clinically stable schizophrenia or schizoaffective disorder (SZ, *n* = 21) and group-matched controls (CT, *n* = 23) (see Table 1 for demographic and clinical details and Supplementary Methods for inclusion/exclusion criteria). This study complied with all relevant ethical regulations for work with human participants; the study protocol was approved by the University of Pennsylvania’s Institutional Review Board and written informed consent was obtained in accordance with IRB guidelines. Participants received a fixed compensation and additional payment based on task performance.

### Study design, clinical assessment, image acquisition, and preprocessing

At the intake visit, participants completed a diagnostic interview and trait measures including self-report questionnaires, interviews, cognitive testing, and behavioral tasks. On scan day, state questionnaires and interviews were administered including the Clinical Assessment Interview for Negative Symptoms (CAINS), which provided our primary measure of clinical amotivation. All imaging data were collected on a 3T Siemens TIM TRIO scanner with a 32-channel head coil, including a T1-weighted structural image and four runs of BOLD images during the effort discounting task, which were preprocessed in FSL. See Supplementary Methods for details of assessment, MRI acquisition, quality assurance, and processing.

### fMRI task paradigm

fMRI activation during effort cost-reward trade-off decisions was probed using a new effort discounting task (EDT, Fig. 1a), adapted from a temporal discounting task^80^ by integrating the reward and effort requirements from our earlier out-of-scanner progressive ratio task (PRT) which demonstrated dimensional associations with amotivation and VS hypofunction^8^.

In each trial, participants chose between lower-effort/lower-reward (EASY) and higher-effort/higher-reward (HARD) options. For example, an item might offer a choice between “200 cents for 10 trials” and “370 cents for 130 trials”. Here the number of effort “trials” referred to the number of required repetitions of the Bigger Number Task (BNT), a simple task requiring choosing the larger of two numbers, used in the prior PRT study^8^ (Fig. 1b). Participants did not complete the effort during the scan but were informed that they would perform one randomly selected choice (and receive the associated reward) after scanning was completed, thus fully isolating the decision phase from effort performance and reward receipt.

Participants completed 200 EDT trials divided across 4 runs. Each trial lasted 4 s, with a jittered crosshair intertrial interval (range 2–20 s, mean 6.1 s). The HARD option parametrically varied reward and effort magnitudes, yielding HARD–EASY differences ranging from 10 to 500 cents and 9 to 1779 BNT trials. Reward and effort were uncorrelated across EDT trials by design. If participants did not make a choice within 4 s for a trial, the task simply moved on to a crosshair followed by the next trial. Each run lasted 504 s (168 TRs). See Supplementary Methods for further task details and considerations.

### Behavioral analysis

EDT behavior was modeled using a linear discount function SV = A-B*E. This equation describes how the subjective value (SV) of a particular reward amount (A) is discounted as the effort (E) needed to obtain it increases. The primary behavioral measure of motivation from the EDT was the estimated B parameter; higher B values indicate a stronger negative impact of effort on subjective value, and hence lower motivation. Beta values were subsequently log_10_ transformed (logB) to provide a normal distribution for group analyses. Behavior was also modeled using exploratory hyperbolic and parabolic discount functions to compare to previous literature on effort and delay discounting^15,35,36,75,81^, along with percentage of hard choices as a model-free measure (see Supplementary Methods).

### fMRI timeseries analysis

We applied five main participant-level timeseries models. The first model included a non-parametric regressor of average response across trials (“task”), capturing the effect of making any choice (between EASY and HARD options). The second model included task (non-parametric) and differential subjective value (SVdiff), a parametric regressor capturing the signed difference in SV between the two trial options (HARD–EASY). SV was tailored for each participant using the above linear discount function. The third model included task and parametric SV of the chosen option only (SVchosen). The fourth model included task, parametric differential effort, and parametric differential reward, in order to separately evaluate reward and effort effects. The fifth model included task, parametric chosen effort, and parametric chosen reward. As range adaptation is reported for BOLD responses to parametric regressors in decision-making tasks, all parametric regressors were z-scored within run^72,82^, and were orthogonalized against the task regressor in each model. The four runs of the task were concatenated and analyzed together^83,84^, primarily to increase variability of the SVchosen regressor, as several participants had little variability in chosen value (i.e., chose all easy options in some task runs). For consistency, concatenated images were used for all analyses; results were similar using fixed effects to combine across runs. Additional confound regressors modeled trials with no subject response, constant regressors for each of the 4 runs, and 6 motion parameters to reduce motion-related artifacts. Given that these models are not fully independent of each other and that secondary models are intended to unpack results from other models, we did not apply a statistical correction for multiple comparisons across these models.

### Group level fMRI analysis

Subject-level contrasts of interest (task, subjective value, reward, effort) were then examined in group analyses, identifying regions where fMRI responses were significant on average, correlated with CAINS amotivation, or reflected categorical group differences (CT > SZ). Random-effects group-level analysis was implemented voxelwise in FSL. Primary analyses focused on the a priori VS ROI, defined using 10 mm spheres around bilateral peak voxels associated with SV in a meta-analysis^55^; dACC and vmPFC were secondary ROIs (see Supplementary Methods and Supplementary Fig. 1). Exploratory whole-brain analyses tested for effects outside of hypothesized regions. Significant clusters within the ROI masks were defined as *p* < 0.05, FWE-corrected using 5000 permutations in FSL’s *randomise*, with threshold-free cluster enhancement (TFCE)^85,86^ to ensure rigorous control of multiple comparisons. All statistical tests utilized two-tailed *p*-values. Except where noted, correlations were calculated using Pearson’s correlations and group comparisons were examined with two-sample *t* tests; normality of key variables (VS activation, amotivation) was confirmed visually and with a Lilliefors test.

### Reporting summary

Further information on research design is available in the Nature Research Reporting Summary linked to this article.

## Supplementary information

Reporting Summary

Supplementary Information

## Data Availability

All data used in this study are available from the authors upon reasonable request.

## Code availability

All code used in this study is available from the authors upon reasonable request.

## References

[1] Fervaha G, Foussias G, Agid O, Remington G (2015). Motivational deficits in early schizophrenia: prevalent, persistent, and key determinants of functional outcome. Schizophrenia Res..

[2] Foussias G, Agid O, Fervaha G, Remington G (2014). Negative symptoms of schizophrenia: clinical features, relevance to real world functioning and specificity versus other CNS disorders. Eur. Neuropsychopharmacol..

[3] Thomas EC, Luther L, Zullo L, Beck AT, Grant PM (2017). From neurocognition to community participation in serious mental illness: the intermediary role of dysfunctional attitudes and motivation. Psychological Med..

[4] Gard DE, Fisher M, Garrett C, Genevsky A, Vinogradov S (2009). Motivation and its relationship to neurocognition, social cognition, and functional outcome in schizophrenia. Schizophrenia Res..

[5] Hanson E, Healey K, Wolf D, Kohler C (2010). Assessment of pharmacotherapy for negative symptoms of schizophrenia. Curr. Psychiatry Rep..

[6] Treadway MT, Buckholtz JW, Schwartzman AN, Lambert WE, Zald DH (2009). Worth the ‘EEfRT’? The effort expenditure for rewards task as an objective measure of motivation and anhedonia. PLoS ONE.

[7] Gold JM, Waltz JA, Frank MJ (2015). Effort cost computation in schizophrenia: a commentary on the recent literature. Biol. Psychiatry.

[8] Wolf DH (2014). Amotivation in schizophrenia: integrated assessment with behavioral, clinical, and imaging measures. Schizophr. Bull..

[9] Green MF, Horan WP, Barch DM, Gold JM (2015). Effort-based decision making: a novel approach for assessing motivation in schizophrenia. Schizophrenia Bull..

[10] Barch DM, Treadway MT, Schoen N (2014). Effort, anhedonia, and function in schizophrenia: reduced effort allocation predicts amotivation and functional impairment. J. Abnorm. Psychol..

[11] Fervaha G, Foussias G, Agid O, Remington G (2013). Amotivation and functional outcomes in early schizophrenia. Psychiatry Res..

[12] Strauss GP (2016). Avolition in schizophrenia is associated with reduced willingness to expend effort for reward on a Progressive Ratio task. Schizophrenia Res..

[13] Gold JM (2013). Negative symptoms of schizophrenia are associated with abnormal effort-cost computations. Biol. Psychiatry.

[14] Culbreth A, Westbrook A, Barch D (2016). Negative symptoms are associated with an increased subjective cost of cognitive effort. J. Abnorm Psychol..

[15] Hartmann MN (2015). Apathy but not diminished expression in schizophrenia is associated with discounting of monetary rewards by physical effort. Schizophr. Bull..

[16] McCarthy JM, Treadway MT, Bennett ME, Blanchard JJ (2016). Inefficient effort allocation and negative symptoms in individuals with schizophrenia. Schizophr. Res..

[17] Wang J (2015). Anhedonia in schizophrenia: deficits in both motivation and hedonic capacity. Schizophrenia Res..

[18] Horan WP (2015). Effort-based decision-making paradigms for clinical trials in schizophrenia: part 2—external validity and correlates. Schizophr. Bull..

[19] Reddy LF (2015). Effort-based decision-making paradigms for clinical trials in schizophrenia: part 1—psychometric characteristics of 5 paradigms. Schizophr. Bull..

[20] Manohar SG, Husain M (2016). Human ventromedial prefrontal lesions alter incentivisation by reward. Cortex.

[21] Takeuchi H (2014). Regional gray matter density is associated with achievement motivation: evidence from voxel-based morphometry. Brain Struct. Funct..

[22] Ming D (2016). Examining brain structures associated with the motive to achieve success and the motive to avoid failure: A voxel-based morphometry study. Soc. Neurosci..

[23] Tye KM (2013). Dopamine neurons modulate neural encoding and expression of depression-related behaviour. Nature.

[24] Parvizi J, Rangarajan V, Shirer WR, Desai N, Greicius MD (2013). The will to persevere induced by electrical stimulation of the human cingulate gyrus. Neuron.

[25] Walton ME, Bouret S, Walton ME (2019). What is the relationship between dopamine and effort?. Trend. Neurosci..

[26] Floresco SB, Ghods-Sharifi S (2007). Amygdala-prefrontal cortical circuitry regulates effort-based decision making. Cereb. Cortex.

[27] Hosking JG, Cocker PJ, Winstanley CA (2016). Prefrontal cortical inactivations decrease willingness to expend cognitive effort on a rodent cost/benefit decision-making task. Cereb. Cortex.

[28] Floresco SB, Tse MT, Ghods-Sharifi S (2008). Dopaminergic and glutamatergic regulation of effort- and delay-based decision making. Neuropsychopharmacology.

[29] Salamone JD, Correa M, Farrar A, Mingote SM (2007). Effort-related functions of nucleus accumbens dopamine and associated forebrain circuits. Psychopharmacology.

[30] Salamone JD, Koychev I, Correa M, McGuire P (2015). Neurobiological basis of motivational deficits in psychopathology. Eur. Neuropsychopharmacol..

[31] Soutschek A (2019). Dopaminergic D1 receptor stimulation affects effort and risk preferences. Biol. Psychiatry.

[32] Bryce CA, Floresco SB (2019). Alterations in effort-related decision-making induced by stimulation of dopamine D1, D2, D3, and corticotropin-releasing factor receptors in nucleus accumbens subregions. Psychopharmacology.

[33] Botvinick MM, Huffstetler S, McGuire JT (2009). Effort discounting in human nucleus accumbens. Cogn. Affect Behav. Neurosci..

[34] McGuire JT, Botvinick MM (2010). Prefrontal cortex, cognitive control, and the registration of decision costs. Proc. Natl Acad. Sci. USA.

[35] Croxson PL, Walton ME, O’Reilly JX, Behrens TE, Rushworth MF (2009). Effort-based cost-benefit valuation and the human brain. J. Neurosci..

[36] Prévost C, Pessiglione M, Météreau E, Cléry-Melin ML, Dreher JC (2010). Separate valuation subsystems for delay and effort decision costs. J. Neurosci..

[37] Krebs RM, Boehler CN, Egner T, Woldorff MG (2011). The neural underpinnings of how reward associations can both guide and misguide attention. J. Neurosci..

[38] Kurniawan IT (2010). Choosing to make an effort: the role of striatum in signaling physical effort of a chosen action. J. Neurophysiol..

[39] Schmidt L, Lebreton M, Cléry-Melin ML, Daunizeau J, Pessiglione M (2012). Neural mechanisms underlying motivation of mental versus physical effort. PLoS Biol..

[40] Aridan N, Malecek NJ, Poldrack RA, Schonberg T (2019). Neural correlates of effort-based valuation with prospective choices. Neuroimage.

[41] Arulpragasam AR, Cooper JA, Nuutinen MR, Treadway MT (2018). Corticoinsular circuits encode subjective value expectation and violation for effortful goal-directed behavior. Proc. Natl Acad. Sci. USA.

[42] Bernacer J (2019). An amygdala-cingulate network underpins changes in effort-based decision making after a fitness program. Neuroimage.

[43] Treadway MT (2012). Dopaminergic mechanisms of individual differences in human effort-based decision-making. J. Neurosci..

[44] Dobryakova E, Jessup RK, Tricomi E (2017). Modulation of ventral striatal activity by cognitive effort. Neuroimage.

[45] Seaman KL (2018). Subjective value representations during effort, probability and time discounting across adulthood. Soc. Cogn. Affect. Neurosci..

[46] Bonnelle V, Manohar S, Behrens T, Husain M (2016). Individual differences in premotor brain systems underlie behavioral apathy. Cereb. Cortex.

[47] Klein-Flugge MC, Kennerley SW, Friston K, Bestmann S (2016). Neural signatures of value comparison in human cingulate cortex during decisions requiring an effort-reward trade-off. J. Neurosci..

[48] Bernacer J (2016). Brain correlates of the intrinsic subjective cost of effort in sedentary volunteers. Prog. Brain Res..

[49] Massar SAA, Libedinsky C, Weiyan C, Huettel SA, Chee MWL (2015). Separate and overlapping brain areas encode subjective value during delay and effort discounting. NeuroImage.

[50] Chong TT (2017). Neurocomputational mechanisms underlying subjective valuation of effort costs. PLoS Biol..

[51] Huang J (2016). Neural substrates of the impaired effort expenditure decision making in schizophrenia. Neuropsychology.

[52] Park IH, Lee BC, Kim J-J, Kim JI, Koo M-S (2017). Effort-based reinforcement processing and functional connectivity underlying amotivation in medicated patients with depression and schizophrenia. J. Neuro..

[53] Culbreth AJ, Moran EK, Kandala S, Westbrook A, Barch DM (2020). Effort, avolition, and motivational experience in schizophrenia: analysis of behavioral and neuroimaging data with relationships to daily motivational experience. Clin. Psychological Sci..

[54] Kable JW, Glimcher PW (2010). An “as soon as possible” effect in human intertemporal decision making: behavioral evidence and neural mechanisms. J. Neurophysiol..

[55] Bartra O, McGuire JT, Kable JW (2013). The valuation system: a coordinate-based meta-analysis of BOLD fMRI experiments examining neural correlates of subjective value. Neuroimage.

[56] Westbrook A, Lamichhane B, Braver T (2019). The subjective value of cognitive effort is encoded by a domain-general valuation network. J. Neurosci..

[57] Juckel G (2006). Dysfunction of ventral striatal reward prediction in schizophrenic patients treated with typical, not atypical, neuroleptics. Psychopharmacology.

[58] de Leeuw M, Kahn RS, Vink M (2015). Fronto-striatal dysfunction during reward processing in unaffected siblings of schizophrenia patients. Schizophr. Bull..

[59] Dowd EC, Barch DM (2012). Pavlovian reward prediction and receipt in schizophrenia: relationship to anhedonia. PLoS ONE.

[60] Radua J (2015). Ventral striatal activation during reward processing in psychosis: a neurofunctional meta-analysis. JAMA Psychiatry.

[61] Wolf DH (2008). Auditory oddball fMRI in schizophrenia: association of negative symptoms with regional hypoactivation to novel distractors. Brain Imaging Behav..

[62] Simon JJ (2010). Neural correlates of reward processing in schizophrenia-relationship to apathy and depression. Schizophr. Res..

[63] Maia TV, Frank MJ (2017). An Integrative perspective on the role of dopamine in schizophrenia. Biol. Psychiatry.

[64] Wolf DH (2011). Striatal intrinsic reinforcement signals during recognition memory: relationship to response bias and dysregulation in schizophrenia. Front. Behav. Neurosci..

[65] Satterthwaite TD (2012). Being right is its own reward: load and performance related ventral striatum activation to correct responses during a working memory task in youth. Neuroimage.

[66] Cuthbert BN (2014). Translating intermediate phenotypes to psychopathology: the NIMH Research Domain Criteria. Psychophysiology.

[67] Hogan PS, Galaro JK, Chib VS (2019). Roles of ventromedial prefrontal cortex and anterior cingulate in subjective valuation of prospective effort. Cereb. Cortex.

[68] Skvortsova V, Palminteri S, Pessiglione M (2014). Learning to minimize efforts versus maximizing rewards: computational principles and neural correlates. J. Neuro.

[69] Wang S, Shi Y, Li BM (2017). Neural representation of cost-benefit selections in rat anterior cingulate cortex in self-paced decision making. Neurobiol. Learn Mem..

[70] Shenhav A, Straccia MA, Cohen JD, Botvinick MM (2014). Anterior cingulate engagement in a foraging context reflects choice difficulty, not foraging value. Nat. Neurosci..

[71] Jimura K, Chushak MS, Westbrook A, Braver TS (2018). Intertemporal decision-making involves prefrontal control mechanisms associated with working memory. Cereb. Cortex.

[72] Lebreton M, Bavard S, Daunizeau J, Palminteri S (2019). Assessing inter-individual differences with task-related functional neuroimaging. Nat. Hum. Behav..

[73] Fervaha G (2013). Incentive motivation deficits in schizophrenia reflect effort computation impairments during cost-benefit decision-making. J. Psychiatr. Res..

[74] Culbreth AJ, Moran EK, Barch DM (2018). Effort-cost decision-making in psychosis and depression: could a similar behavioral deficit arise from disparate psychological and neural mechanisms?. Psychol. Med..

[75] Cooper JA (2019). Effortful goal-directed behavior in schizophrenia: computational subtypes and associations with cognition. J. Abnorm Psychol..

[76] Bailey MR, Chun E, Schipani E, Balsam PD, Simpson EH (2020). Dissociating the effects of dopamine D2 receptors on effort-based versus value-based decision making using a novel behavioral approach. Behav. Neurosci..

[77] Terenzi D, Mainetto E, Barbato M, Rumiati RI, Aiello M (2019). Temporal and effort cost decision-making in healthy individuals with subclinical psychotic symptoms. Sci. Rep..

[78] Chang WC (2019). Effort-based decision-making impairment in patients with clinically-stabilized first-episode psychosis and its relationship with amotivation and psychosocial functioning. Eur. Neuropsychopharmacol..

[79] Gupta T, Cowan HR, Strauss GP, Walker EF, Mittal VA (2020). Deconstructing negative symptoms in individuals at clinical high-risk for psychosis: evidence for volitional and diminished emotionality subgroups that predict clinical presentation and functional outcome. Schizophr. Bull..

[80] Senecal N, Wang T, Thompson E, Kable JW (2012). Normative arguments from experts and peers reduce delay discounting. Judgm. Decis. Mak..

[81] Kurniawan IT, Guitart-Masip M, Dayan P, Dolan RJ (2013). Effort and valuation in the brain: the effects of anticipation and execution. J. Neuro.

[82] Cox KM, Kable JW (2014). BOLD subjective value signals exhibit robust range adaptation. J. Neurosci..

[83] MacKillop J (2012). The neuroeconomics of nicotine dependence: a preliminary functional magnetic resonance imaging study of delay discounting of monetary and cigarette rewards in smokers. Psychiatry Res..

[84] Manning J (2014). Personality influences temporal discounting preferences: behavioral and brain evidence. Neuroimage.

[85] Smith SM, Nichols TE (2009). Threshold-free cluster enhancement: addressing problems of smoothing, threshold dependence and localisation in cluster inference. Neuroimage.

[86] Eklund A, Nichols TE, Knutsson H (2016). Cluster failure: Why fMRI inferences for spatial extent have inflated false-positive rates. Proc. Natl Acad. Sci. USA.

