# Supplementary material for: Relationship of ventral striatum activation during effort discounting to clinical amotivation severity in schizophrenia
Source: NPJ Schizophr. 2021 Oct 8;7:48. doi: 10.1038/s41537-021-00178-9 (PMC8501117; doi:10.1038/s41537-021-00178-9)
Supplement: Supplementary file 1 — Reporting Summary [file 41537_2021_178_MOESM1_ESM.pdf]

nature portfolio

Corresponding author(s): Greer Prettyman and Daniel Wolf

Last updated by author(s): Aug 5, 2021

Reporting Summary

Nature Portfolio wishes to improve the reproducibility of the work that we publish. This form provides structure for consistency and transparency in reporting. For further information on Nature Portfolio policies, see our Editorial Policies and the Editorial Policy Checklist.

Statistics

For all statistical analyses, confirm that the following items are present in the figure legend, table legend, main text, or Methods section.

n/a	Confirmed	
☐	☒	The exact sample size (n) for each experimental group/condition, given as a discrete number and unit of measurement
☐	☒	A statement on whether measurements were taken from distinct samples or whether the same sample was measured repeatedly
☐	☒	The statistical test(s) used AND whether they are one- or two-sided<br>*Only common tests should be described solely by name; describe more complex techniques in the Methods section.*
☐	☒	A description of all covariates tested
☐	☒	A description of any assumptions or corrections, such as tests of normality and adjustment for multiple comparisons
☐	☒	A full description of the statistical parameters including central tendency (e.g. means) or other basic estimates (e.g. regression coefficient) AND variation (e.g. standard deviation) or associated estimates of uncertainty (e.g. confidence intervals)
☐	☒	For null hypothesis testing, the test statistic (e.g. F, t, r) with confidence intervals, effect sizes, degrees of freedom and P value noted<br>*Give P values as exact values whenever suitable.*
☒	☐	For Bayesian analysis, information on the choice of priors and Markov chain Monte Carlo settings
☒	☐	For hierarchical and complex designs, identification of the appropriate level for tests and full reporting of outcomes
☐	☒	Estimates of effect sizes (e.g. Cohen's d, Pearson's r), indicating how they were calculated

Our web collection on statistics for biologists contains articles on many of the points above.

Software and code

Policy information about availability of computer code

Data collection	E-Prime 2.0, RedCap, Oracle, 3T Siemens TIM TRIO scanner, WebCNP
Data analysis	FSL FEAT 6.0, Matlab R2020b, R Studio 1.1.383

For manuscripts utilizing custom algorithms or software that are central to the research but not yet described in published literature, software must be made available to editors and reviewers. We strongly encourage code deposition in a community repository (e.g. GitHub). See the Nature Portfolio guidelines for submitting code & software for further information.

Data

Policy information about availability of data

All manuscripts must include a data availability statement. This statement should provide the following information, where applicable:
- Accession codes, unique identifiers, or web links for publicly available datasets
- A description of any restrictions on data availability
- For clinical datasets or third party data, please ensure that the statement adheres to our policy

All data used in this study are available from the authors upon reasonable request.

Field-specific reporting

Please select the one below that is the best fit for your research. If you are not sure, read the appropriate sections before making your selection.

☐ Life sciences ☒ Behavioural & social sciences ☐ Ecological, evolutionary & environmental sciences

For a reference copy of the document with all sections, see nature.com/documents/nr-reporting-summary-flat.pdf

Behavioural & social sciences study design

All studies must disclose on these points even when the disclosure is negative.

Study description	Quantitative experimental
Research sample	Of 50 enrolled participants, 44 had analyzable data, including individuals with clinically stable schizophrenia or schizoaffective disorder (SZ, n=21) and group-matched controls (CT, n=23). All participants were between the ages 18-55, proficiency in English language, and ability to understand study procedures and provide informed consent. This study sample was chosen to study effort discounting in patients with schizophrenia and a control group of healthy individuals. Participants in our sample were representative of these clinical groups.
Sampling strategy	The sample size was chosen based on available data from SZ and CT participants who completed an in-scanner effort discounting task. Sample sizes were determined on the basis of prior fMRI neuroeconomic studies in SZ and our sample sizes are consistent with similar prior published work in this area.
Data collection	Participants completed intake and scan visits, with a Research Assistant present. At the intake visit, participants completed a diagnostic interview and trait measures including self-report questionnaires, interviews, cognitive testing, and behavioral tasks. On scan day, state questionnaires and interviews were administered, as well as a 3T MRI scan. A University of Pennsylvania MRI technician was present at each scan in addition to the participant and Research Assistant. Interview data was collected with pen and paper and later inputted into digital archives for storage and analysis. Self-report questionnaires were collected using REDCAP software. Demographic and medication data was collected and directly input into Oracle. Scanner performance data and performance on the behavioral PRT was directly logged in E-Prime. This was not an interventional study, and researchers were not blind to diagnostic group or study hypotheses.
Timing	Data was collected from 2012 through 2014.
Data exclusions	Fifty participants were enrolled. Six individuals were enrolled but excluded from analyses. Two CT participants completed intake visits but did not return for scans. One participant with SZ experienced claustrophobia in the scanner. Two individuals (1 CT, 1 SZ) had positive results for pre-scan urine drug screens and were excluded. One participant with SZ was excluded because they chose the easy option on every single EDT trial which precluded parametric chosen value analyses, and they were also a >4SD outlier in VS activation in multiple contrasts (no other participants were >=3SD). Our final sample for imaging analyses thus included 44 individuals (21 SZ, 23 CT).
Non-participation	Two CT participants completed intake visits but did not return for scans. These participants were excluded from analyses.
Randomization	This was not an interventional study so participants were not randomized to experimental groups. Participants for the two diagnostic groups (healthy control, schizoprhenia) were identified using structured clinical interview. Effects of controlling for potential confound covariates were examined in exploratory confound/sensitivity analyses.

Reporting for specific materials, systems and methods

We require information from authors about some types of materials, experimental systems and methods used in many studies. Here, indicate whether each material, system or method listed is relevant to your study. If you are not sure if a list item applies to your research, read the appropriate section before selecting a response.

Materials & experimental systems

n/a	Involved in the study	
☒	☐	Antibodies
☒	☐	Eukaryotic cell lines
☒	☐	Palaeontology and archaeology
☒	☐	Animals and other organisms
☐	☒	Human research participants
☐	☒	Clinical data
☒	☐	Dual use research of concern

Methods

n/a	Involved in the study	
☒	☐	ChIP-seq
☒	☐	Flow cytometry
☐	☒	MRI-based neuroimaging

Human research participants

Policy information about studies involving human research participants

Population characteristics	See above
Recruitment	Patients with schizophrenia as well as healthy controls were recruited through the Psychosis and Neurodevelopment Section/ Schizophrenia Research Center at the Hospital of the University of Pennsylvania. The Center maintains a database of research participants who have agreed to be contacted for studies and recruits new participants from local mental health facilities as well as advertisements. There is a potential for self-selection bias given the volunteer nature of this study, so we cannot rule out biases in terms of characteristics of patients and healthy controls who volunteer to participate in research. We do not expect this to substantially impact our results.
Ethics oversight	The study was approved by the University of Pennsylvania's Institutional Review Board.

Note that full information on the approval of the study protocol must also be provided in the manuscript.

Clinical data

Policy information about clinical studies

All manuscripts should comply with the ICMJE guidelines for publication of clinical research and a completed CONSORT checklist must be included with all submissions.

Clinical trial registration	n/a not a clinical trial
Study protocol	n/a not a clinical trial
Data collection	n/a not a clinical trial
Outcomes	n/a not a clinical trial

Magnetic resonance imaging

Experimental design

Design type	Task-fMRI, event-related
Design specifications	Participants completed 200 EDT trials divided across 4 runs. Each trial lasted 4 seconds, with a jittered crosshair intertrial interval (range 2s-20s, mean 6.1s). The HARD option parametrically varied reward and effort magnitudes, yielding HARD-EASY differences ranging from 10-500 cents and 9-1779 Bigger Number Test trials. Reward and effort were uncorrelated across EDT trials by design. If participants did not make a choice within 4 seconds for a trial, the task simply moved on to a crosshair followed by the next trial. Each run lasted 504s (168 TRs). In two of the task runs the EASY option was fixed at 200 cents for 10 effort trials, and in the other two runs the EASY option provided 0 cents for 1 effort trial; order was counterbalanced (first/last runs vs. middle two runs).
Behavioral performance measures	Button presses, response time, and choice were recorded in E-Prime behavior logfiles. EDT behavior was modeled using a linear discount function SV=A-B*E. This equation describes how the subjective value (SV) of a particular reward amount (A) is discounted as the effort (E) needed to obtain it increases. The primary behavioral measure of motivation from the EDT was the estimated B parameter; higher B values indicate a stronger negative impact of effort on subjective value, and hence lower motivation. Beta values were subsequently log10 transformed (logB) to provide a normal distribution for group analyses. Behavior was also modeled using exploratory hyperbolic and parabolic discount functions to compare to previous literature on effort and delay discounting, along with percentage of hard choices as a model-free measure. Mean, SD, and one sample t tests were used to ensure that participants were making choices as expected and two sample t tests and Pearson's correlations were used to test a priori hypotheses about group differences and clinical correlations related to task behavior.

Acquisition

Imaging type(s)	Functional and structural
Field strength	3 Tesla
Sequence & imaging parameters	All imaging data were collected on a 3T Siemens TIM TRIO scanner (Erlangen, Germany) with a 32-channel head coil. A T1-weighted structural image was acquired for use in co-registration (MPRAGE, TR=1810 ms, TE= 3.51 ms, TI=1100 ms, flip angle 9, FOV= 240 x 180 mm, matrix= 256 × 192, slices = 160, slice/skip thickness = 1 mm/0 mm). Four runs of BOLD images during the effort discounting task were acquired using a whole-brain, 2-D echo-planar sequence (TR/TE=3000/30 ms, flip angle 90°, FOV=192 mm, matrix= 64X64, slice thickness/gap=3/0mm, 45 slices axial-oblique -30° from AC-PC line).
Area of acquisition	Whole brain

Diffusion MRI ☐ Used ☒ Not used

Preprocessing

Preprocessing software	fMRI preprocessing was performed using standard algorithms in FSL including distortion correction using a B0 magnetic field map, slice-time correction, motion correction, 6mm spatial smoothing, high-pass filtering (120s) and co-registration to the MPRAGE. Subject-level time-series analyses employed general linear models (GLM) in FSL's FEAT version 6.0, using a canonical double-gamma hemodynamic response function for convolution.
Normalization	Normalization/Registration was linear. The MNI152_T1_2mm_brain template was used with 12 degrees of freedom for group standardized space
Normalization template	The MNI152_T1_2mm_brain template was used for group standardized space.
Noise and artifact removal	Motion correction was done with MCFLIRT in FEAT during preprocessing. We included 6 motion parameters in all first level models to reduce motion-related artifacts.
Volume censoring	We did not use volume censoring and instead included motion parameters as regressors in all first level models.

Statistical modeling & inference

Model type and settings	First levels models used mixed effects: FLAME 1. We applied five main participant-level timeseries models. The first model included a non-parametric regressor of average response across trials ("task"), capturing the effect of making any choice (between EASY and HARD options).The second model included task (non-parametric) and differential subjective value (SVdiff), a parametric regressor capturing the signed difference in SV between the two trial options (HARD-EASY). SV was tailored for each participant using the above linear discount function. The third model included task and parametric SV of the chosen option only (SVchosen). The fourth model included task, parametric differential effort, and parametric differential reward, in order to separately evaluate reward and effort effects. The fifth model included task, parametric chosen effort, and parametric chosen reward. Random-effects group-level analysis was implemented voxelwise in FSL.
Effect(s) tested	Subject-level contrasts of interest (task, subjective value, reward, effort) were examined in group analyses, identifying regions where fMRI responses were significant on average, correlated with CAINS amotivation, or reflected categorical group differences (CT>SZ). Random-effects group-level analysis was implemented voxelwise in FSL.

Specify type of analysis: ☐ Whole brain ☐ ROI-based ☒ Both

Anatomical location(s)	Primary analyses focused on the a priori VS ROI; dACC and vmPFC were secondary ROIs. All ROIs were created by forming 10mm spheres around peak coordinates identified in a meta-analysis of subjective value (Bartra et al. 2013). Bilateral VS was centered on left peak MNI coordinates -12,12,-6 and right peak 12,10,-6; dACC on -2,28,28; and vmPFC on 2,46,-8.<br>Exploratory whole-brain analyses tested for effects outside of hypothesized regions.

Statistic type for inference<br>(See Eklund et al. 2016)	Random-effects group-level analysis was implemented voxelwise in FSL.
Correction	Significant clusters within the ROI masks were defined as p<0.05, FWE-corrected using 5000 permutations in FSL's randomise, with threshold-free cluster enhancement (TFCE) to ensure rigorous control of multiple comparisons.

Models & analysis

n/a	Involved in the study
☒	☐ Functional and/or effective connectivity
☒	☐ Graph analysis
☒	☐ Multivariate modeling or predictive analysis
