# Supplementary material for: Relationship of ventral striatum activation during effort discounting to clinical amotivation severity in schizophrenia
Source: NPJ Schizophr. 2021 Oct 8;7:48. doi: 10.1038/s41537-021-00178-9 (PMC8501117; doi:10.1038/s41537-021-00178-9)
Supplement: Supplementary file 2 — Supplementary Information [file 41537_2021_178_MOESM2_ESM.pdf]

SUPPLEMENTARY INFORMATION

Relationship of ventral striatum activation during effort discounting to clinical amotivation severity in schizophrenia

Authors: Greer E. Prettyman[1], Joseph W. Kable[3], Paige Didier[1], Sheila Shankar[1], Theodore D. Satterthwaite[1,5,6], Christos Davatzikos[2,5], Warren B. Bilker[4], Mark A. Elliott[2], Kosha Ruparel[1], Daniel H. Wolf[1,5]

[1] Department of Psychiatry, University of Pennsylvania, Philadelphia PA 19104, USA
[2] Department of Radiology, University of Pennsylvania, Philadelphia PA 19104, USA
[3] Department of Psychology, University of Pennsylvania, Philadelphia PA 19104, USA
[4] Department of Biostatistics, Epidemiology & Informatics, University of Pennsylvania, Philadelphia PA 19104, USA
[5] Center for Biomedical Image Computing and Analytics, University of Pennsylvania, Philadelphia PA 19104, USA
[6] Penn Lifespan Informatics and Neuroimaging Center, University of Pennsylvania, Philadelphia PA 19104, USA

Contents of Supplementary Information:

Supplementary Methods. Inclusions/exclusions; assessment; MRI acquisition, preprocessing and quality assurance; region-of interest (ROI) definitions; task design, rationale and instructions; neuroeconomic modeling

Supplementary Discussion. Summary of imaging findings; secondary behavioral measures; whole-brain analysis of task contrast; spatial selectivity of group difference in VS activation; spatial selectivity of chosen value vs. differential value; confound/sensitivity analyses

Supplementary Table 1. Trial-wise description of effort discounting task (EDT)

Supplementary Table 2. VS and amotivation relationship to potential confound variables

Supplementary Table 3. Group and amotivation effects in VS, controlling for potential confounds

Supplementary Figure 1. Regions of Interest

Supplementary Figure 2. Whole-brain task-activated regions

Supplementary Figure 3. Whole-brain task-deactivated regions

Supplementary Figure 4. VS-selectivity of group difference

Supplementary Figure 5. VS-selectivity of CAINS amotivation correlation

Supplementary Figure 6. Spatial comparison of subjective value (SV) models

Supplementary References

SUPPLEMENTARY METHODS

Participant Inclusions and Exclusions

Patients with schizophrenia (SZ) as well as healthy controls (CT) were recruited through the Psychosis and Neurodevelopment Section/Schizophrenia Research Center at the Hospital of the University of Pennsylvania. The Center maintains a database of research participants who have agreed to be contacted for studies and recruits new participants from local mental health facilities as well as advertisements.

Enrollment inclusion criteria were age 18-55, proficiency in English language, and ability to understand study procedures and provide informed consent. Patients were included if they had a diagnosis of schizophrenia, schizoaffective, or schizophreniform disorder, and were clinically stable without acute safety risks or recent (< 2 week) changes in psychiatric medication regimen. Exclusions for all participants included metallic implants or other MRI contraindications; significant medical or neurological illness expected to impact brain function or impede participation; pervasive developmental disorder or intellectual disability; substance use disorder (except nicotine) within past six months or failure of drug screen reflecting recent use and risk of intoxication/withdrawal; pregnancy. Healthy controls were excluded if diagnostic assessment revealed a history of an Axis I or Axis II psychiatric disorder or a first-degree family history of psychosis.

Fifty participants were enrolled. Six individuals were enrolled but excluded from analyses. Two CT participants completed intake visits but did not return for scans. One participant with SZ experienced claustrophobia in the scanner. Two individuals (1 CT, 1 SZ) had positive results for

pre-scan urine drug screens and were excluded. One participant with SZ was excluded because they chose the easy option on every single EDT trial which precluded parametric chosen value analyses, and they were also a >4SD outlier in VS activation in multiple contrasts (no other participants were >=3SD). No participants met our exclusion cutoff for excessive in-scanner motion (mean relative displacement [MRD] > 0.3mm). Our final sample for imaging analyses thus included 44 individuals (21 SZ, 23 CT).

Participant Assessment

Diagnostic assessment utilized the Structured Clinical Interview for DSM-IV Axis I Disorders (SCID)[1], and family history was assessed with the Family Interview for Genetic Studies (FIGS)[2]. The Clinical Assessment Interview for Negative Symptoms (CAINS beta version)[3] was used as the primary measure of clinical amotivation and other negative symptoms. In the CAINS each item is measured on a 5-point scale (0-4) with higher numbers indicating increasing levels of severity. Individual negative symptom domain scores were calculated by averaging across relevant items. For CAINS amotivation, the key clinical measure in this study, scores were averaged across social, vocational, and recreational motivation subdomains (three social motivation items were first averaged together before averaging with vocational and recreational motivation items to provide equal weighting of these subdomains). The CAINS was originally designed to be able to assess motivation as a distinct domain, as suggested by the NIMH consensus conference on negative symptoms[4]. Based on this, we also designed our study to examine CAINS amotivation as a distinct domain. The Scale for the Assessment of Negative Symptoms (SANS)[5] was collected as a secondary measure to allow the negative symptom severity of this sample to be compared to samples in prior literature. For the SANS, the four

domains (alogia, flat affect, avolition-apathy, anhedonia-asociality) were measured using their respective global items, and the overall SANS score calculated as the average of these global items (the attention subscale was not utilized).

At the intake visit, participants completed a Premorbid Adjustment Scale (PAS) interview[6] and a battery of trait self-report measures. On the day of the scan, the research coordinator administered the CAINS interview and participants completed the additional self-report scales. Research coordinators were trained to administer the CAINS interview, achieving an inter-rater reliability criterion of >= 0.8.

Overall positive symptom severity was measured by averaging the five global items from the Scale for the Assessment of Positive Symptoms (SAPS)[7]. Depression was assessed using the Calgary Depression Scale for Schizophrenia (CDSS)[8], and summarized as the total of all 9 individual items. Nicotine dependence was assessed using the Fagerstrom Test for Nicotine Dependence (FTND)[9]. Socioeconomic status (SES) was estimated with a weighted combination score of education and occupation using the Hollingshead approach[10].

Global cognitive performance was based on the Penn Computerized Neurocognitive Battery (CNB)[11] and calculated by averaging z-scores (normalized across all participants) from the following domains (tests): episodic memory (word memory), working memory (letter n-back), emotion identification, language (verbal reasoning by analogy), attention (continuous performance test), abstraction and mental flexibility (conditional exclusion test). Participants completed additional behavioral tasks to assess hypothetical intertemporal discounting[12] and risk

discounting adapted from Yu et al. 2017[13], as well as a behavioral progressive ratio task[14]. Antipsychotic medication dosages in estimated chlorpromazine equivalents were determined with conversion calculations applied routinely in our research center. For first-generation antipsychotics, these are based on Davis' 1976 article[15] and for second/third-generation antipsychotics based on Kohler et al.[16] as follows: chlorpromazine 100mg = olanzapine 4mg = risperidone 1.2 mg = quetiapine 140 mg = ziprasidone 32 mg = aripiprazole 6mg = clozapine 100mg. Of the 21 patients who completed the study, 17 were taking second-generation antipsychotics only, 3 first-generation antipsychotics only, and 1 was taking no antipsychotics. Two patients were being treated with 2 atypical antipsychotics, but all others received antipsychotic monotherapy. The specific antipsychotic medications were olanzapine (6), clozapine (5), risperidone (4), aripiprazole (3), lurasidone (1), haloperidol (1), perphenazine (1), and trifluoperazine (1). Other medications used by the patients included antidepressants (6), benzodiazepines (4), anticholinergics (2), other psychotropics (3); none were on lithium, anticonvulsants, or stimulants.

MRI Image Acquisition

All imaging data were collected on a 3T Siemens TIM TRIO scanner (Erlangen, Germany) with a 32-channel head coil. A T1-weighted structural image was acquired for use in co-registration (MPRAGE, TR=1810 ms, TE= 3.51 ms, TI=1100 ms, flip angle 9, FOV= 240 x 180 mm, matrix= 256 × 192, slices = 160, slice/skip thickness = 1 mm/0 mm). Four runs of BOLD images during the effort discounting task were acquired using a whole-brain, 2-D echo-planar sequence (TR/TE=3000/30 ms, flip angle 90°, FOV=192 mm, matrix= 64X64, slice

thickness/gap=3/0mm, 45 slices axial-oblique -30° from AC-PC line). Responses and reaction times were recorded with a fiberoptic two-button response pad.

fMRI Preprocessing

fMRI preprocessing was performed using standard algorithms in FSL including distortion correction using a B0 magnetic field map, slice-time correction, motion correction, 6mm spatial smoothing, high-pass filtering (120s) and co-registration to the MPRAGE. Subject-level time-series analyses employed general linear models (GLM) in FSL's FEAT version 6.0, using a canonical double-gamma hemodynamic response function for convolution.

fMRI Quality Assurance

All imaging data were collected by a trained MRI research coordinator together with University of Pennsylvania MRI technicians. At the time of scan acquisition, all structural and functional images were visually inspected for artifact, subject motion or operator error. The temporal SNR of BOLD sequences was monitored across studies in our center to confirm proper scanner function. During pre-processing, visual inspection confirmed expected spatial coverage and registration for each BOLD run in MNI template space. As head motion is usually the major source of artifact in fMRI data, motion parameters were included as confounds in the timeseries analysis, and mean relative displacement was examined as a covariate in group-level sensitivity analysis; no participants met our exclusion threshold of MRD>0.3mm.

Regions of Interest (ROIs)

All ROIs were created by forming 10mm spheres around peak coordinates identified in a meta-analysis of subjective value[17] (See **Supplementary Fig. 1**). Bilateral VS was centered on left peak MNI coordinates -12,12,-6 and right peak 12,10,-6; dACC on -2,28,28; and vmPFC on 2,46,-8.

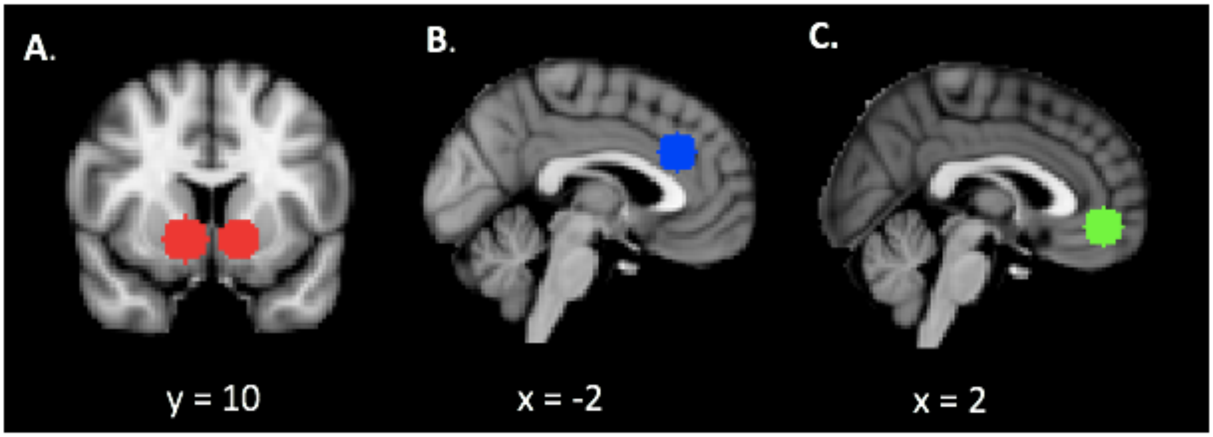

Supplementary Fig. **1** ROIs used for analysis. Bilateral ventral striatum (VS, **A**) was the primary ROI, while dorsal anterior cingulate cortex (dACC, **B**) and ventromedial prefrontal cortex (vmPFC, **C**) were secondary.

Effort Discounting Task (EDT) Design & Rationale

We developed our EDT by adapting and integrating two prior tasks. One was a delay discounting task (DDT) successfully applied in prior behavioral[13,18] and fMRI[19] studies. We adopted the DDT's general structure in terms of number of runs, number and timing of trials, and stimulus/choice format, with orthogonal parametric variation of reward and delay in the more delayed option, and parametric variation of reward in the immediate option. The other task was an out-of-scanner progressive ratio task (PRT[14]) which demonstrated dimensional correlations

with CAINS amotivation and with VS activation during a reward-guessing fMRI task. The PRT reward varied from 10 to 50 cents and required effort varied from 1 to 500 trials of the Bigger Number Task (BNT, main Fig. 1b), spanning cent-per-effort ratios ranging from 0.1 (high motivation) to 10 (low motivation). For our EDT, we used the BNT for our effort trials, and selected reward and effort values to span a similar range of effort discounting beta parameters as the PRT (the EDT beta parameter is the estimated slope of the linear discounting function, and also corresponds to a ratio of cents/trials). Unlike the PRT where the participant made each choice and then performed the effort (if chosen), the EDT was designed to focus on isolating activation during decision phase. Thus, each EDT trial consisted of only evaluating the options and deciding between them, without performing the effort. This ensured that the timing of fMRI trials was the same across participants and avoided the increase in task-correlated motion artifact that normally arises with any repetitive or effortful motion. Additionally, VS activation is increased by preparation for effort exertion[20] and we wanted to fully separate effort decisions from effort exertion to avoid obscuring the valuation effect[21]. In order to encourage participants to choose according to their true preferences without producing undue burden, a single trial from the 200 decision trials was selected at random to be performed after scanning. The ordering of options was pseudorandomized across trials, unlike the progressive PRT, to avoid expectation effects and to decorrelate any low temporal frequency fMRI effects ("scanner drift"). The parametric reward and effort amounts of the HARD option were orthogonalized by design, allowing these two components to be jointly modeled to distinguish effects of monetary reward and effort cost. As reward and effort for the EASY option were kept constant within a single run, this approach also decorrelated differential (HARD-EASY) subjective value, a standard approach in the temporal discounting literature.

In two of the task runs the EASY option was fixed at 200 cents for 10 BNT effort trials, and in the other two runs the EASY option provided 0 cents for 1 effort trial; order was counterbalanced (first/last runs vs. middle two runs). We included these two run types in order to encourage attention to the details of both EASY and HARD options (which were left-right counterbalanced), and to increase the range of differential monetary reward tested while keeping the HARD reward options the same across runs. We did not use 0 effort for any EASY option, as we wanted to ensure the participant knew they would need to engage at least minimally with the effort task after the scan.

We were not able to design the task to decorrelate reward and effort effects on chosen SV, because these depend on participant-specific choices. Chosen SV, chosen effort, and chosen reward all tend to correlate across trials, because people will choose the high effort option only when the reward is sufficiently high to produce a positive subjective value.

Some of the features of our design are different from most prior effort discounting fMRI paradigms. One, mentioned above, is that we do not include any effort performance during the scan itself. Another is that we use a wide range of HARD reward and effort. HARD effort varied 10-1780 BNT trials, and HARD reward varied 200-500 cents, yielding HARD-EASY differences across runs ranging from 10-500 cents and 9-1779 trials (see Supplementary Table 1 below). The high range of reward and effort in our design is thus quite salient, in contrast to many prior effort discounting paradigms which use a relatively low and restricted range of reward or effort. This wide range is expected to enhance detection of parametric effects on brain

activation, as well as to increase participant engagement with the task. The robust full-sample effects we see for parametric fMRI contrasts suggests this wide range is effective on average; however, it is possible that wide-range parametric regressors are more sensitive to trial-wise variation in noise or scaling effects, which could negatively impact individual difference analyses.

The inclusion of high effort levels also makes the SV of the hard choice (and thus the differential SV) take on negative values on many trials for most participants. This has important implications for modeling, as the hyperbolic model (or the similarly-shaped exponential model) typically used in temporal discounting do not allow SV to be negative, and therefore are particularly unlikely to provide optimal modeling in a task like ours (see Supplementary Discussion below for data demonstrating this). To the extent that hyperbolic modeling in prior effort discounting work was successful, it likely reflected the use of maximal efforts that were not especially costly. The high effort and resulting negative SVs also leads to strong differences between differential SV (HARD-EASY) and chosen SV, as the former will often be negative while the latter is almost always positive. Whether having many negative SV hard options shifts the extent to which participants (and their brain valuation circuits) are sensitized to differential SV vs. chosen SV is unknown, but could contribute to the relative spatial selectivity of accumbens activation for chosen SV vs. differential SV (see Supplementary Fig. 6 below).

Given the interest in the field in understanding whether cognitive and physical effort discounting exhibit distinct neurobehavioral characteristics, it is worth commenting on our EDT in terms of the nature of the effort. The Bigger Number Task was expressly designed within the original

PRT context to involve very low levels of both cognitive and physical demands. The goal was to ensure that performance would not be significantly influenced by cognitive or motor impairment which are common in schizophrenia, and to have the task capture motivational aspects that were general rather than specific to cognition or physical effort. Consistent with that goal, neither cognitive ability nor reaction time related to motivation in that PRT study[14]. Nonetheless, some experts in the field categorize our task as a cognitive effort task[22,23]. Due to the above considerations, we do not agree with this categorization when used to imply a clear distinction from physical effort tasks, although our task clearly has a cognitive component. To date we do not have the empirical data to determine whether our task "behaves" more like a cognitive task, or more like a physical task, or is indeed domain general as intended.

A few minor design flaws were discovered during analysis. First, a single trial in one run with a HARD effort value of 10 trials was in some participants paired with an EASY trial with the same effort requirement, leading to a differential effort of zero; this trial was excluded from behavioral and imaging analysis. Second, the final trial in each run was late enough in the run that its expected hemodynamic response occurred after the run ended; this trial was also excluded from analysis. Third, due to a technical error, only $0 EASY runs were administered for one included participant (SZ), and only $2 EASY runs were administered to another included participant (CT).

					RUN ORDER TYPE 1							RUN ORDER TYPE 2						
Trial #	Run	Onset (s)	Crosshair Duration (s)	Easy On Right	HARD Cents	HARD Trials	EASY Cents	EASY Trials	DIFF Cents	DIFF Trials	Beta-Equiv	HARD Cents	HARD Trials	EASY Cents	EASY Trials	DIFF Cents	DIFF Trials	Beta-Equiv
1	1	-13	5	0	230	210	0	1	230	209	1.100	230	210	200	10	30	200	0.150
2	1	-4	5	1	280	50	0	1	280	49	5.714	280	50	200	10	80	40	2.000
3	1	5	5	1	310	550	0	1	310	549	0.565	310	550	200	10	110	540	0.204
4	1	26	17	1	390	340	0	1	390	339	1.150	390	340	200	10	190	330	0.576
5	1	38	8	0	250	70	0	1	250	69	3.623	250	70	200	10	50	60	0.833
6	1	44	2	1	230	450	0	1	230	449	0.512	230	450	200	10	30	440	0.068
7	1	50	2	1	380	1490	0	1	380	1489	0.255	380	1490	200	10	180	1480	0.122
8	1	65	11	1	410	970	0	1	410	969	0.423	410	970	200	10	210	960	0.219
9	1	77	8	0	320	680	0	1	320	679	0.471	320	680	200	10	120	670	0.179
10	1	83	2	1	470	480	0	1	470	479	0.981	470	480	200	10	270	470	0.574
11	1	89	2	0	320	480	0	1	320	479	0.668	320	480	200	10	120	470	0.255
12	1	107	14	0	280	530	0	1	280	529	0.529	280	530	200	10	80	520	0.154
13	1	125	14	1	430	1610	0	1	430	1609	0.267	430	1610	200	10	230	1600	0.144
14	1	131	2	1	480	1070	0	1	480	1069	0.449	480	1070	200	10	280	1060	0.264
15	1	143	8	0	420	150	0	1	420	149	2.819	420	150	200	10	220	140	1.571
16	1	152	5	0	390	480	0	1	390	479	0.814	390	480	200	10	190	470	0.404
17	1	167	11	1	260	1490	0	1	260	1489	0.175	260	1490	200	10	60	1480	0.041
18	1	173	2	0	210	840	0	1	210	839	0.250	210	840	200	10	10	830	0.012
19	1	179	2	0	380	1780	0	1	380	1779	0.214	380	1780	200	10	180	1770	0.102
20	1	185	2	0	320	70	0	1	320	69	4.638	320	70	200	10	120	60	2.000
21	1	191	2	1	260	840	0	1	260	839	0.310	260	840	200	10	60	830	0.072
22	1	197	2	0	340	70	0	1	340	69	4.928	340	70	200	10	140	60	2.333
23	1	203	2	0	210	1610	0	1	210	1609	0.131	210	1610	200	10	10	1600	0.006
24	1	215	8	1	420	70	0	1	420	69	6.087	420	70	200	10	220	60	3.667
25	1	236	17	1	280	480	0	1	280	479	0.585	280	480	200	10	80	470	0.170
26	1	245	5	0	290	40	0	1	290	39	7.436	290	40	200	10	90	30	3.000
27	1	251	2	1	330	550	0	1	330	549	0.601	330	550	200	10	130	540	0.241
28	1	260	5	0	480	430	0	1	480	429	1.119	480	430	200	10	280	420	0.667
29	1	272	8	0	330	1070	0	1	330	1069	0.309	330	1070	200	10	130	1060	0.123
30	1	278	2	1	230	1780	0	1	230	1779	0.129	230	1780	200	10	30	1770	0.017
31	1	284	2	1	500	1240	0	1	500	1239	0.404	500	1240	200	10	300	1230	0.244
32	1	290	2	0	300	340	0	1	300	339	0.885	300	340	200	10	100	330	0.303
33	1	299	5	1	270	70	0	1	270	69	3.913	270	70	200	10	70	60	1.167
34	1	323	20	1	260	1240	0	1	260	1239	0.210	260	1240	200	10	60	1230	0.049
35	1	329	2	1	390	1240	0	1	390	1239	0.315	390	1240	200	10	190	1230	0.154
36	1	338	5	0	260	1070	0	1	260	1069	0.243	260	1070	200	10	60	1060	0.057
37	1	347	5	1	370	320	0	1	370	319	1.160	370	320	200	10	170	310	0.548
38	1	353	2	0	420	1780	0	1	420	1779	0.236	420	1780	200	10	220	1770	0.124
39	1	362	5	1	450	1490	0	1	450	1489	0.302	450	1490	200	10	250	1480	0.169
40	1	368	2	0	440	1240	0	1	440	1239	0.355	440	1240	200	10	240	1230	0.195
41	1	377	5	0	290	680	0	1	290	679	0.427	290	680	200	10	90	670	0.134
42	1	392	11	0	290	170	0	1	290	169	1.716	290	170	200	10	90	160	0.563
43	1	401	5	0	270	290	0	1	270	289	0.934	270	290	200	10	70	280	0.250
44	1	413	8	0	480	230	0	1	480	229	2.096	480	230	200	10	280	220	1.273
45	1	431	14	0	310	970	0	1	310	969	0.320	310	970	200	10	110	960	0.115
46	1	437	2	1	440	340	0	1	440	339	1.298	440	340	200	10	240	330	0.727
47	1	452	11	1	480	840	0	1	480	839	0.572	480	840	200	10	280	830	0.337
48	1	464	8	1	480	340	0	1	480	339	1.416	480	340	200	10	280	330	0.848
49	1	473	5	0	230	1240	0	1	230	1239	0.186	230	1240	200	10	30	1230	0.024
50	1	482	5	1	280	1240	0	1	280	1239	0.226	280	1240	200	10	80	1230	0.065
1	2	-13	5	0	230	210	200	10	30	200	0.150	230	210	0	1	230	209	1.100
2	2	-4	5	1	260	150	200	10	60	140	0.429	260	150	0	1	260	149	1.745
3	2	5	5	1	210	970	200	10	10	960	0.010	210	970	0	1	210	969	0.217
4	2	11	2	0	220	1070	200	10	20	1060	0.019	220	1070	0	1	220	1069	0.206
5	2	17	2	1	490	470	200	10	290	460	0.630	490	470	0	1	490	469	1.045
6	2	26	5	0	480	290	200	10	280	280	1.000	480	290	0	1	480	289	1.661
7	2	44	14	1	370	1780	200	10	170	1770	0.096	370	1780	0	1	370	1779	0.208
8	2	50	2	0	410	680	200	10	210	670	0.313	410	680	0	1	410	679	0.604
9	2	59	5	1	310	840	200	10	110	830	0.133	310	840	0	1	310	839	0.369
10	2	68	5	0	230	290	200	10	30	280	0.107	230	290	0	1	230	289	0.796
11	2	74	2	0	480	680	200	10	280	670	0.418	480	680	0	1	480	679	0.707
12	2	86	8	0	350	680	200	10	150	670	0.224	350	680	0	1	350	679	0.515
13	2	92	2	0	370	1610	200	10	170	1600	0.106	370	1610	0	1	370	1609	0.230
14	2	101	5	0	250	510	200	10	50	500	0.100	250	510	0	1	250	509	0.491
15	2	116	11	0	410	1610	200	10	210	1600	0.131	410	1610	0	1	410	1609	0.255
16	2	140	20	0	500	680	200	10	300	670	0.448	500	680	0	1	500	679	0.736
17	2	149	5	1	380	340	200	10	180	330	0.545	380	340	0	1	380	339	1.121
18	2	161	8	1	380	290	200	10	180	280	0.643	380	290	0	1	380	289	1.315
19	2	170	5	1	450	110	200	10	250	100	2.500	450	110	0	1	450	109	4.128
20	2	176	2	0	230	480	200	10	30	470	0.064	230	480	0	1	230	479	0.480
21	2	182	2	0	380	680	200	10	180	670	0.269	380	680	0	1	380	679	0.560
22	2	191	5	1	450	1070	200	10	250	1060	0.236	450	1070	0	1	450	1069	0.421
23	2	209	14	0	410	1240	200	10	210	1230	0.171	410	1240	0	1	410	1239	0.331
24	2	215	2	1	430	180	200	10	230	170	1.353	430	180	0	1	430	179	2.402
25	2	221	2	1	280	840	200	10	80	830	0.096	280	840	0	1	280	839	0.334
26	2	227	2	1	490	1780	200	10	290	1770	0.164	490	1780	0	1	490	1779	0.275
27	2	236	5	0	350	840	200	10	150	830	0.181	350	840	0	1	350	839	0.417
28	2	248	8	1	230	1490	200	10	30	1480	0.020	230	1490	0	1	230	1489	0.154
29	2	254	2	0	250	1780	200	10	50	1770	0.028	250	1780	0	1	250	1779	0.141
30	2	272	14	1	480	480	200	10	280	470	0.596	480	480	0	1	480	479	1.002
31	2	281	5	1	290	1070	200	10	90	1060	0.085	290	1070	0	1	290	1069	0.271
32	2	287	2	1	490	970	200	10	290	960	0.302	490	970	0	1	490	969	0.506
33	2	302	11	1	420	840	200	10	220	830	0.265	420	840	0	1	420	839	0.501
34	2	308	2	1	490	840	200	10	290	830	0.349	490	840	0	1	490	839	0.584
35	2	317	5	0	280	290	200	10	80	280	0.286	280	290	0	1	280	289	0.969
36	2	329	8	0	380	520	200	10	180	510	0.353	380	520	0	1	380	519	0.732
37	2	335	2	0	440	70	200	10	240	60	4.000	440	70	0	1	440	69	6.377
38	2	344	5	1	330	1780	200	10	130	1770	0.073	330	1780	0	1	330	1779	0.185
39	2	350	2	0	410	260	200	10	210	250	0.840	410	260	0	1	410	259	1.583
40	2	365	11	1	360	480	200	10	160	470	0.340	360	480	0	1	360	479	0.752
41	2	371	2	1	380	1070	200	10	180	1060	0.170	380	1070	0	1	380	1069	0.355
42	2	377	2	1	230	480	200	10	30	470	0.064	230	480	0	1	230	479	0.480
43	2	398	17	1	270	340	200	10	70	330	0.212	270	340	0	1	270	339	0.796
44	2	404	2	0	220	680	200	10	20	670	0.030	220	680	0	1	220	679	0.324
45	2	425	17	0	310	140	200	10	110	130	0.846	310	140	0	1	310	139	2.230
46	2	437	8	0	290	1490	200	10	90	1480	0.061	290	1490	0	1	290	1489	0.195
47	2	449	8	1	480	1490	200	10	280	1480	0.189	480	1490	0	1	480	1489	0.322
48	2	455	2	0	490	1490	200	10	290	1480	0.196	490	1490	0	1	490	1489	0.329
49	2	467	8	0	460	340	200	10	260	330	0.788	460	340	0	1	460	339	1.357
50	2	482	11	1	230	70	200	10	30	60	0.500	230	70	0	1	230	69	3.333

1	3	-13	5	0	320	450	200	10	120	440	0.273	320	450	0	1	320	449	0.713
2	3	-4	5	1	260	140	200	10	60	130	0.462	260	140	0	1	260	139	1.871
3	3	2	2	0	250	260	200	10	50	250	0.200	250	260	0	1	250	259	0.965
4	3	23	17	0	320	1070	200	10	120	1060	0.113	320	1070	0	1	320	1069	0.299
5	3	29	2	0	330	290	200	10	130	280	0.464	330	290	0	1	330	289	1.142
6	3	38	5	0	370	1070	200	10	170	1060	0.160	370	1070	0	1	370	1069	0.346
7	3	50	8	1	210	340	200	10	10	330	0.030	210	340	0	1	210	339	0.619
8	3	59	5	0	230	1490	200	10	30	1480	0.020	230	1490	0	1	230	1489	0.154
9	3	74	11	1	470	250	200	10	270	240	1.125	470	250	0	1	470	249	1.888
10	3	83	5	0	480	80	200	10	280	70	4.000	480	80	0	1	480	79	6.076
11	3	89	2	1	340	340	200	10	140	330	0.424	340	340	0	1	340	339	1.003
12	3	95	2	0	320	1610	200	10	120	1600	0.075	320	1610	0	1	320	1609	0.199
13	3	104	5	0	420	1490	200	10	220	1480	0.149	420	1490	0	1	420	1489	0.282
14	3	110	2	1	210	160	200	10	10	150	0.067	210	160	0	1	210	159	1.321
15	3	119	5	1	240	1240	200	10	40	1230	0.033	240	1240	0	1	240	1239	0.194
16	3	134	11	0	370	280	200	10	170	270	0.630	370	280	0	1	370	279	1.326
17	3	140	2	1	250	1070	200	10	50	1060	0.047	250	1070	0	1	250	1069	0.234
18	3	146	2	1	350	170	200	10	150	160	0.938	350	170	0	1	350	169	2.071
19	3	152	2	1	330	1240	200	10	130	1230	0.106	330	1240	0	1	330	1239	0.266
20	3	158	2	1	460	70	200	10	260	60	4.333	460	70	0	1	460	69	6.667
21	3	164	2	1	360	680	200	10	160	670	0.239	360	680	0	1	360	679	0.530
22	3	176	8	0	490	430	200	10	290	420	0.690	490	430	0	1	490	429	1.142
23	3	185	5	0	480	1610	200	10	280	1600	0.175	480	1610	0	1	480	1609	0.298
24	3	191	2	0	320	290	200	10	120	280	0.429	320	290	0	1	320	289	1.107
25	3	200	5	0	230	90	200	10	30	80	0.375	230	90	0	1	230	89	2.584
26	3	212	8	1	370	500	200	10	170	490	0.347	370	500	0	1	370	499	0.741
27	3	221	5	1	230	130	200	10	30	120	0.250	230	130	0	1	230	129	1.783
28	3	245	20	0	390	149	200	10	190	139	1.367	390	149	0	1	390	148	2.635
29	3	254	5	1	370	970	200	10	170	960	0.177	370	970	0	1	370	969	0.382
30	3	272	14	0	290	520	200	10	90	510	0.176	290	520	0	1	290	519	0.559
31	3	284	8	1	480	1780	200	10	280	1770	0.158	480	1780	0	1	480	1779	0.270
32	3	293	5	0	340	480	200	10	140	470	0.298	340	480	0	1	340	479	0.710
33	3	305	8	1	290	70	200	10	90	60	1.500	290	70	0	1	290	69	4.203
34	3	326	17	0	440	360	200	10	240	350	0.686	440	360	0	1	440	359	1.226
35	3	344	14	1	420	290	200	10	220	280	0.786	420	290	0	1	420	289	1.453
36	3	350	2	1	460	1070	200	10	260	1060	0.245	460	1070	0	1	460	1069	0.430
37	3	356	2	1	440	290	200	10	240	280	0.857	440	290	0	1	440	289	1.522
38	3	365	5	0	330	1490	200	10	130	1480	0.088	330	1490	0	1	330	1489	0.222
39	3	371	2	0	430	970	200	10	230	960	0.240	430	970	0	1	430	969	0.444
40	3	386	11	1	310	340	200	10	110	330	0.333	310	340	0	1	310	339	0.914
41	3	392	2	1	250	1610	200	10	50	1600	0.031	250	1610	0	1	250	1609	0.155
42	3	404	8	1	350	1610	200	10	150	1600	0.094	350	1610	0	1	350	1609	0.218
43	3	410	2	0	290	1780	200	10	90	1770	0.051	290	1780	0	1	290	1779	0.163
44	3	416	2	0	480	290	200	10	280	280	1.000	480	290	0	1	480	289	1.661
45	3	431	11	1	420	460	200	10	220	450	0.489	420	460	0	1	420	459	0.915
46	3	449	14	0	240	680	200	10	40	670	0.060	240	680	0	1	240	679	0.353
47	3	455	2	0	410	480	200	10	210	470	0.447	410	480	0	1	410	479	0.856
48	3	464	5	0	410	340	200	10	210	330	0.636	410	340	0	1	410	339	1.209
49	3	476	8	1	280	430	200	10	80	420	0.190	280	430	0	1	280	429	0.653
50	3	482	2	1	270	680	200	10	70	670	0.104	270	680	0	1	270	679	0.398
1	4	-13	5	1	320	700	0	1	320	699	0.458	320	700	200	10	120	690	0.174
2	4	-4	5	0	280	440	0	1	280	439	0.638	280	440	200	10	80	430	0.186
3	4	2	2	0	260	30	0	1	260	29	8.966	260	30	200	10	60	20	3.000
4	4	14	8	1	440	600	0	1	440	599	0.735	440	600	200	10	240	590	0.407
5	4	23	5	0	490	80	0	1	490	79	6.203	490	80	200	10	290	70	4.143
6	4	35	8	0	470	1610	0	1	470	1609	0.292	470	1610	200	10	270	1600	0.169
7	4	41	2	0	260	160	0	1	260	159	1.635	260	160	200	10	60	150	0.400
8	4	47	2	1	400	70	0	1	400	69	5.797	400	70	200	10	200	60	3.333
9	4	59	8	0	460	970	0	1	460	969	0.475	460	970	200	10	260	960	0.271
10	4	68	5	0	270	970	0	1	270	969	0.279	270	970	200	10	70	960	0.073
11	4	83	11	1	380	80	0	1	380	79	4.810	380	80	200	10	180	70	2.571
12	4	89	2	0	380	970	0	1	380	969	0.392	380	970	200	10	180	960	0.188
13	4	98	5	1	250	840	0	1	250	839	0.298	250	840	200	10	50	830	0.060
14	4	104	2	0	290	370	0	1	290	369	0.786	290	370	200	10	90	360	0.250
15	4	113	5	0	400	1610	0	1	400	1609	0.249	400	1610	200	10	200	1600	0.125
16	4	119	2	0	240	290	0	1	240	289	0.830	240	290	200	10	40	280	0.143
17	4	125	2	1	250	340	0	1	250	339	0.737	250	340	200	10	50	330	0.152
18	4	143	14	0	290	970	0	1	290	969	0.299	290	970	200	10	90	960	0.094
19	4	149	2	0	400	280	0	1	400	279	1.434	400	280	200	10	200	270	0.741
20	4	170	17	0	360	290	0	1	360	289	1.246	360	290	200	10	160	280	0.571
21	4	179	5	0	310	1780	0	1	310	1779	0.174	310	1780	200	10	110	1770	0.062
22	4	185	2	1	230	460	0	1	230	459	0.501	230	460	200	10	30	450	0.067
23	4	197	8	1	280	1780	0	1	280	1779	0.157	280	1780	200	10	80	1770	0.045
24	4	203	2	1	360	840	0	1	360	839	0.429	360	840	200	10	160	830	0.193
25	4	221	14	0	430	480	0	1	430	479	0.898	430	480	200	10	230	470	0.489
26	4	230	5	0	390	30	0	1	390	29	13.448	390	30	200	10	190	20	9.500
27	4	239	5	0	440	1780	0	1	440	1779	0.247	440	1780	200	10	240	1770	0.136
28	4	251	8	1	340	970	0	1	340	969	0.351	340	970	200	10	140	960	0.146
29	4	257	2	0	380	70	0	1	380	69	5.507	380	70	200	10	180	60	3.000
30	4	269	8	1	310	370	0	1	310	369	0.840	310	370	200	10	110	360	0.306
31	4	275	2	1	330	1240	0	1	330	1239	0.266	330	1240	200	10	130	1230	0.106
32	4	296	17	1	290	1610	0	1	290	1609	0.180	290	1610	200	10	90	1600	0.056
33	4	305	5	0	260	480	0	1	260	479	0.543	260	480	200	10	60	470	0.128
34	4	323	14	1	250	400	0	1	250	399	0.627	250	400	200	10	50	390	0.128
35	4	329	2	1	310	1490	0	1	310	1489	0.208	310	1490	200	10	110	1480	0.074
36	4	344	11	1	420	330	0	1	420	329	1.277	420	330	200	10	220	320	0.688
37	4	359	11	0	320	230	0	1	320	229	1.397	320	230	200	10	120	220	0.545
38	4	365	2	1	360	1240	0	1	360	1239	0.291	360	1240	200	10	160	1230	0.130
39	4	389	20	1	430	680	0	1	430	679	0.633	430	680	200	10	230	670	0.343
40	4	395	2	0	420	1070	0	1	420	1069	0.393	420	1070	200	10	220	1060	0.208
41	4	404	5	0	330	10	0	1	330	9	36.667	330	10	200	10	130	0	NA
42	4	410	2	1	460	1240	0	1	460	1239	0.371	460	1240	200	10	260	1230	0.211
43	4	422	8	0	260	1610	0	1	260	1609	0.162	260	1610	200	10	60	1600	0.038
44	4	437	11	1	480	480	0	1	480	479	1.002	480	480	200	10	280	470	0.596
45	4	446	5	1	380	320	0	1	380	319	1.191	380	320	200	10	180	310	0.581
46	4	455	5	0	240	970	0	1	240	969	0.248	240	970	200	10	40	960	0.042
47	4	461	2	1	330	430	0	1	330	429	0.769	330	430	200	10	130	420	0.310
48	4	470	5	1	390	840	0	1	390	839	0.465	390	840	200	10	190	830	0.229
49	4	476	2	1	480	70	0	1	480	69	6.957	480	70	200	10	280	60	4.667
50	4	482	2	1	450	840	0	1	450	839	0.536	450	840	200	10	250	830	0.301

Notes:

First two trials in each run with negative onset times were from deleted portion of BOLD run, not analyzed

Crosshair Duration refers to the fixation period preceding that trial

Easy On Right (1 if EASY option on right) refers to left-right counterbalancing of options

Beta-Equiv refers to the linear model beta value which is equivalent for the HARD and EASY option

Supplementary Table 1. Trial-wise details of EDT task.

EDT Task Instructions

Prior to the scanning session, each participant completed a brief pre-scan practice of the EDT task. A research coordinator read all instructions to the participant. If the participant made any "irrational" choices (ex. 5000 BNT trials for 5 cents) then the coordinator re-emphasized that they should choose only trials they would actually be willing to do, since any one of the trials could be chosen at random for them to do after the scan. The participant was also asked to explain the task back to the coordinator in their own words to ensure their thorough understanding of the task prior to completion during fMRI.

During the scan, the following task instructions appeared on the screen and were simultaneously read aloud to the participant by the study administrator:

Earlier you completed the Bigger Number Task, pressing the key for the bigger number over many trials. Now in this Choice Task you will be asked to choose between two offers. One option will be no money for completing a single trial (or one key press) of the Bigger Number Task. The other option will offer more money for completing more trials of the Bigger Number Task.

For example, if the screen shows the following [example screen displayed] then you are being asked to choose between getting $4.90 for 50 trials of pressing the key for the bigger number or $0.00 for 1 trial of pressing the key for the bigger number. You will make many such choices. One of your choices will be picked at random for you to complete after the scan has ended. Since any of your choices might be selected to be completed, you should answer every question as if you were going to have to complete the option you choose.

Neuroeconomic Modeling

Our primary discounting model used a linear discounting function for reasons described above. In order to assess our *a priori* expectation that a linear discounting model would provide the best fit to the EDT behavioral data, we also examined the behavioral results for hyperbolic and parabolic discounting functions, both of which have been used in prior effort discounting literature [24-31]. The linear discounting model used the equation SV=A-B*E. We compared this to hyperbolic equation SV=A/(1+B*E) and parabolic equation SV=A-B*E^2.

B is estimated by fitting a logistic regression that assumes the subject's choices are a stochastic function of the difference in subjective value between the two options. Estimation was performed using standard function minimization routines in MATLAB, as in Yu et al. 2017[13]. An additional free scaling parameter σ estimates the probability of a particular choice given this difference in subjective value, according to the equation: $P1 = 1/(1+e^{-\sigma(SV1-SV2)})$, P2 = 1 – P1, where P1 is the probability that the participant chose option 1, and P2 is the probability that the participant chose option 2. The range of the estimated beta parameter was restricted to the range captured by the trials with lowest beta-equivalent and the highest beta-equivalent, where beta-equivalent is the beta which would make the SV of the HARD and EASY option equal. Betas outside this range captured by actual trial options could not be accurately estimated by the model.

SUPPLEMENTARY DISCUSSION

Secondary Behavioral Measures

Behavioral choices (HARD vs. EASY) demonstrated expected patterns on average across all participants. Selection of the harder option progressively increased when it was associated with increasing reward and progressively decreased in association with increasing effort. As the *a priori* SV model does not separate reward and effort effects but rather integrates them, we assessed their separate effects using a logistic regression that included two predictors of hard choice: differential reward (HARD-EASY) and differential effort (HARD-EASY). This yielded t-statistics for reward and effort for each participant which were analyzed at the group level. The positive effect of differential reward in increasing hard choices was highly significant (full sample mean±SD t-value 3.04±1.98, one-sample group t-test t=10.18 p<0.001). The negative effect of differential effort in reducing hard choices was even more significant (full sample mean±SD t-value -3.65±2.20, one-sample group t-test t=-11.18 p<0.001). These effects were highly significant in both CT and SZ groups, although somewhat less robust in SZ (reward CT mean±SD t-value 3.61±2.06, one-sample group t-test t=8.41 p<0.001; reward SZ mean±SD t-value 2.41±1.73, one-sample group t-test t=6.40 p<0.001; effort CT mean±SD t-value -4.23±1.74, one-sample group t-test t=-11.65 p<0.001; effort SZ mean±SD t-value -3.02±2.49, one-sample group t-test t=-5.54 p<0.001). There were no significant correlations of these reward and effort effect measures with CAINS amotivation across the full sample or within SZ (p's >0.1).

Patients and controls did not differ significantly on percent of hard choices made, a secondary measure of behavioral motivation (CT 41±26%, SZ 47±37%, t=-0.64, p=0.53). Percent hard choices did not correlate with clinical amotivation across all participants (r=-0.03, p=0.84) or in

SZ patients alone (r=-0.07, p=0.76), although the direction of the relationship was negative as expected.

We also examined model fit for our *a priori* linear neuroeconomic model of subjective value, as well as for hyperbolic and parabolic models used in the literature (see equations above). Model fit was assessed with the Akaike Information Criteria (AIC) from subject-level discounting models of choice behavior; lower AIC values reflect better model fit, and an AIC difference of >=2 is considered to reflect a significant difference in goodness-of-fit. AIC correlated very strongly with the model R^2 as well as with the percentage of a participant's choices correctly predicted by the model (inverse correlation, r's >0.95), so we did not examine those other metrics in detail. As expected, across the full sample the *a priori* linear model fit behavioral data better than the hyperbolic or parabolic models (linear AIC mean±SD 88±51; hyperbolic 139±65; parabolic 246±886). The average poor fit and high variance for the parabolic model was driven by 3 individuals with >3SD outlier levels of AIC (no such outliers present for linear or hyperbolic); after removing these individuals from analysis the parabolic fit (AIC 98±59) was better than hyperbolic and closer to the linear fit. This model fit ordering linear > parabolic > hyperbolic was confirmed by paired t-tests: linear > hyperbolic fit t=-4.93 p<0.001(negative value is better as lower AIC is better); linear > parabolic t=-2.68 p=0.01, parabolic > hyperbolic t=-3.46 p=0.001. We did not hypothesize group differences or amotivation correlations in model fit but examined these as this could impact model-based results. There were no significant differences in AIC model fit between CT and SZ (linear t=0.94, p=0.35; hyperbolic t=0.27, p=0.79; parabolic t=1.11, p=0.27). There were no significant correlations between linear model fit and CAINS amotivation across the full sample (linear r=-0.02, p=0.92; hyperbolic r=0.03,

p=0.83; parabolic r=-0.07, p=0.65) or within just SZ (linear r=0.05, p=0.84; hyperbolic r=0.07, p=0.76; parabolic r=-0.03 p=0.91). When examining individual participant model fits, the majority of individuals (21 of 44) were best fit by the linear model, while 6 were best fit by hyperbolic, 6 best fit by parabolic, 5 fit equally well by all three models, 4 fit best/equally by linear and parabolic, 2 fit best/equally by linear and hyperbolic. Therefore 33 of 44 participants (75%) had choice patterns best or equally well fit by our *a priori* linear model. Thus, on average across the full sample, task behavior showed the expected pattern consistent with effort-discounted subjective value. However, contrary to expectation, the estimated discounting parameters and percent hard choices were not abnormal in SZ and did not correlate with amotivation severity. In our prior study using the progressive ratio task with similar reward and effort ranges, the relationship between behavioral and clinical amotivation was more robust[14]. This raises the possibility that the greater abstractness of the effort discounting task (e.g., random trial ordering and deferred effort performance) is more vulnerable to cognitive confounds or variation in "task strategy" than the more concrete PRT. Rather than only exhibiting a reduction in willingness to exert effort, some individuals with schizophrenia likely allocate effort in suboptimal ways[26,32], and this may be exacerbated in more complex or abstract tasks. The presence of some across-subject variability in the best-fitting SV model supports this possibility. In addition, our linear model assumes sensitivity to effort cost (hence discounting) is independent of reward levels, and violation of this assumption could impact the accuracy of our behavioral modeling.

The fact that our fMRI task contrast proved more sensitive to clinical effects than our behavioral measures supports the utility of incorporating fMRI in this context, and may reflect a variety of factors impacting behavioral importance that are not captured by our fMRI task-activation

measure. This may also contribute to the lack of clinical correlation with parametric fMRI measures. However, it is important to emphasize that variation in model fit did not confound our fMRI results since model fit did not relate to diagnosis or amotivation. Furthermore, differences in behavioral strategy are unlikely to have impacted our key fMRI results obtained with the model-free task contrast which simply averaged brain response across all trials.

Whole-brain Analysis of Task Contrast

Exploratory whole-brain analysis showed the ROI effects reported in main text and revealed additional task-activated (frontal, occipital and parietal cortices, bilateral insula, thalamus, and task-deactivated (temporal, parietal and medial default-mode) areas (see Supplementary Figs. 2 and 3).

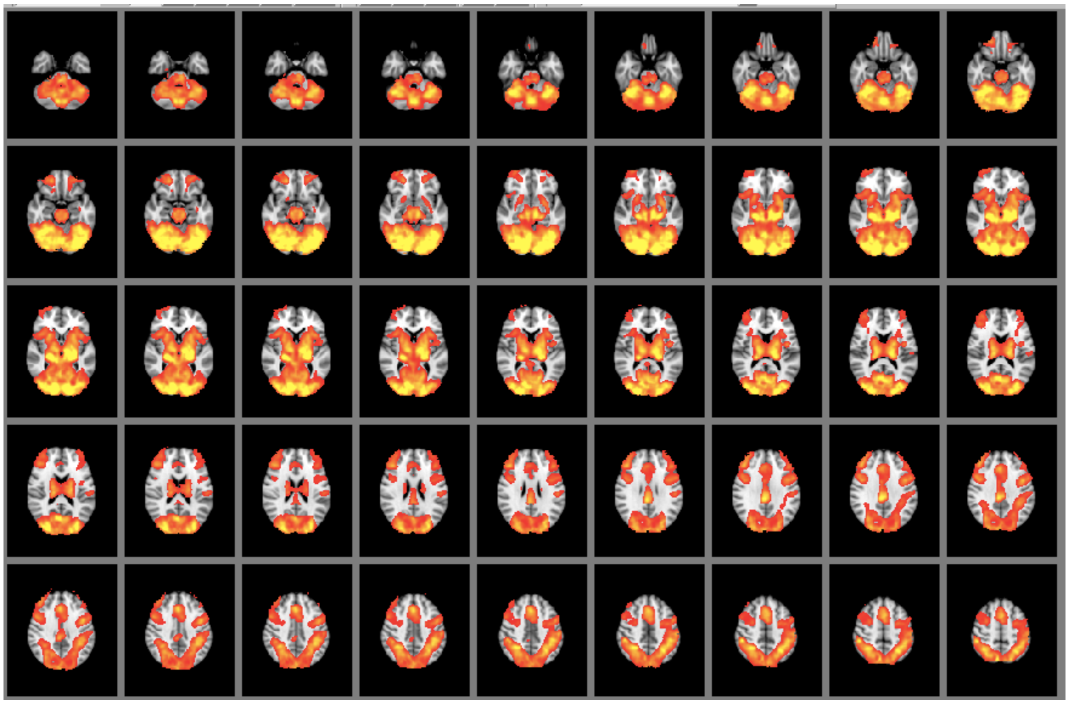

Supplementary Fig. 2. Task-activated regions. Threshold t=3 (red) to 10 (yellow); slices z=-36 to +52 shown. Right on left.

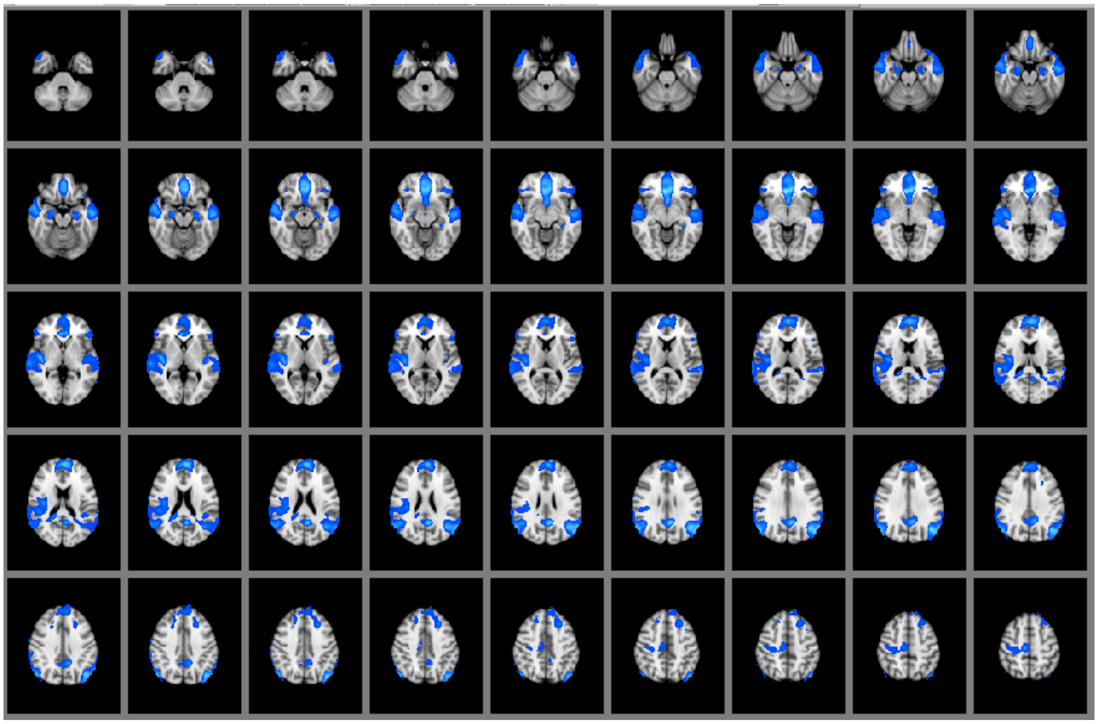

Supplementary Fig. 3. Task-deactivated regions. Threshold t=3 (dark blue) to 10 (bright blue); slices z=-36 to +52 shown. Right on left

VS-selectivity of Clinical Abnormalities in Task Activation

Exploratory whole brain analysis of the task contrast highlighted the selective CT>SZ group difference in the ventral striatum. VS showed the most robust activation of any gray matter region (See Supplementary Fig. 4). The across-sample correlation of task-activation with CAINS amotivation (controlling for group) was also most significant in VS (See Supplementary Fig. 5; whole-brain peak t=-5.23, coordinates 6, 16, 2). While most prior fMRI literature (see references in main text) supports the view that amotivation and other negative symptoms are mainly associated with hypofunction in VS, a few studies do identify this relationship in dorsal striatum[33-35], so evaluating these effects in the whole-brain analysis is of interest. As seen in Supplementary Figure 4, the categorical group effect is not significant in dorsal striatum even at a liberal uncorrected threshold. For the full-sample CAINS correlation seen in Supplementary

Figure 5 (and a similar striatal pattern is seen in the SZ-only CAINS correlation map), the correlation in DS is significant and only slightly less strong than that in VS. It is not clear why the categorical effect might be more VS-selective (vs. DS) than the dimensional effect. Unexamined factors that are more related to the group difference than to amotivation might impact VS more than DS, or perhaps there are different nonlinearities in the amotivation:activation relationship such that "binarizing" the dimensional feature into "high vs. low" categories (similar to SZ vs. CT) retains more sensitivity in VS than in DS.

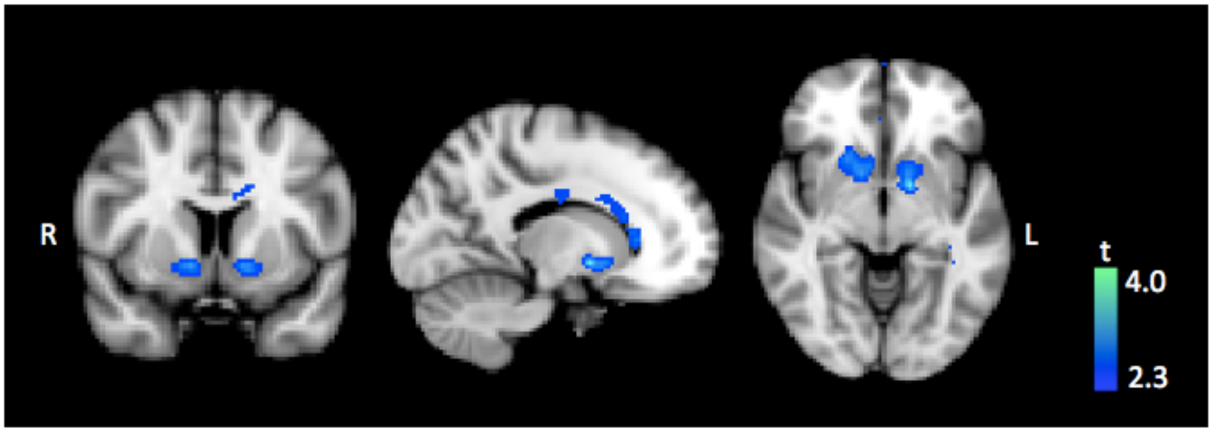

Supplementary Fig. 4. Whole brain SZ < CT group difference to task activation, unmasked and shown at a liberal threshold (t>2.3) to demonstrate selective group difference in bilateral VS relative to other brain regions. Displayed at x=-14, y=8, z=-6.

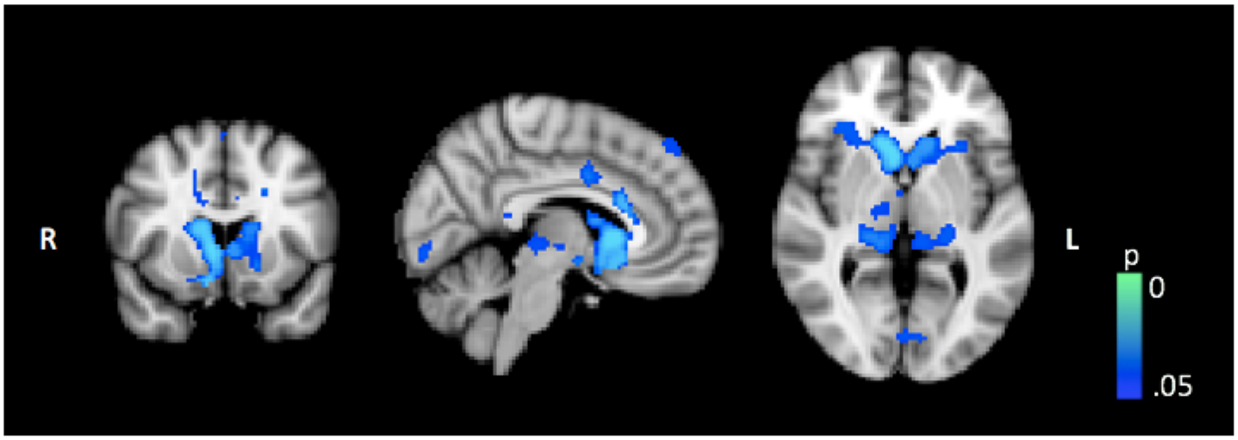

Supplementary Fig. 5. Cluster-corrected whole-brain activation during task correlated with clinical amotivation across all participants, demonstrating strongest effects in VS. Displayed at x=6, y=12, z=2.

Spatial Selectivity of Chosen Value vs. Differential Value

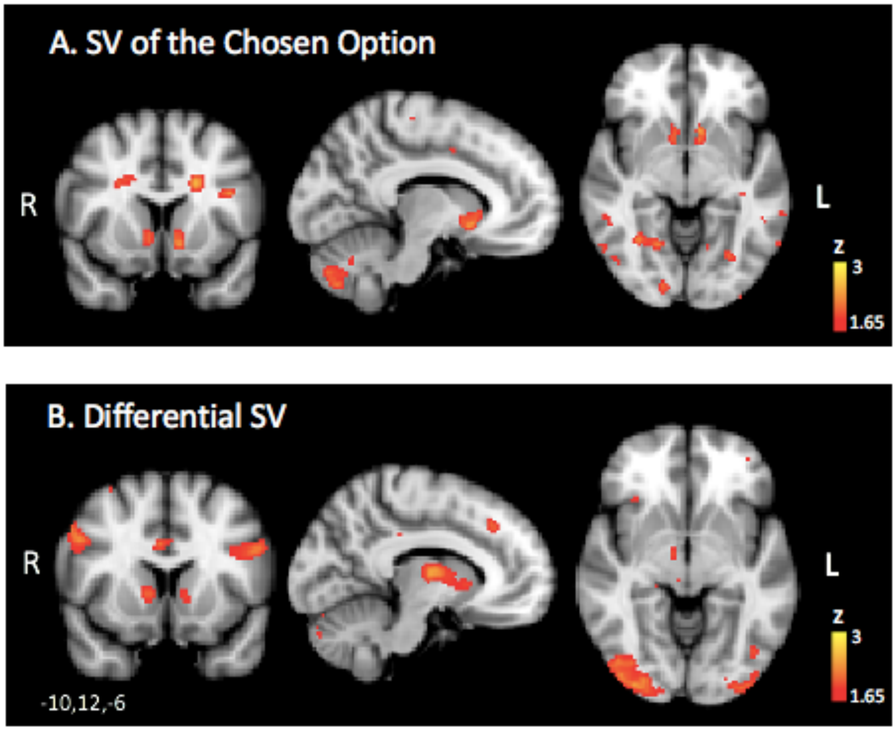

Supplementary Fig. 6. Spatially z-scored contrast maps for mean subjective value of the chosen option (**A**) and mean relative subjective value between the two options (**B**). Images are set at z=1.65 threshold to illustrate differences in the spatial pattern of activation within different subregions of ventral striatum. Compared to the differential SV model, the chosen SV model elicited activation that was more selective for VS, as well as more centered in ventromedial (NAc) positive-valence vs. valence-insensitive salience regions of VS[17]. Images displayed at MNI coordinate -6,10,-6.

Confound and Sensitivity Analyses

There were no significant group differences between SZ and CT in age (t=-1.61 p=0.12), sex (43% female in both groups, Fisher's exact p>=0.99), education level (t=1.83, p=0.07), parental education (t=1.91, p=0.06), smoking (CT 9% smokers, SZ 24% smokers, Fisher's exact p=0.23), in-scanner motion (MRD, t=-0.56 p=0.58), or task reaction time (across-trial median, t=-0.19, p=0.85). There were expected group differences in cognition on the CNB (t=4.42, p<0.001) and all clinical symptoms including CDSS depression (t=-4.12, p<0.001), SAPS global positive symptoms (t=-4.27, p<0.001), CAINS amotivation (t=-4.57, p<0.001), CAINS anhedonia (t=-2.68, p<0.001), and CAINS expressivity (t=-4.05, p<0.001). Note that positive t values indicate CT>SZ for all comparisons.

Relationship with Amotivation

Variable	Full Sample		Within SZ	
	r/t[1]	p	r/t	p
Age	0.06	0.68	-0.47	0.03
Sex[2]	-0.37	0.71	0.21	0.83
Education	-0.39	0.008	-0.30	0.19
Parental Education	-0.42	0.004	-0.26	0.25
Smoking[3]	1.04	0.30	0.47	0.64
In-Scanner Motion	0.10	0.51	-0.09	0.69
Reaction Time	0.13	0.41	0.20	0.38
Cognition	-0.23	0.13	0.26	0.26
Depression	0.42	0.004	0.23	0.32
Anhedonia	0.60	<0.001	0.44	0.05
Expressivity	0.51	<0.001	0.31	0.17
Positive Symptoms	0.57	<0.001	0.49	0.02
Antipsychotic Dose	n/a	n/a	0.09	0.70

Relationship with Ventral Striatum

Variable	Full Sample		Within SZ	
	r/t[1]	p	r/t	p
Age	-0.21	0.17	0.21	0.35
Sex[2]	0.60	0.55	0.03	0.98
Education	0.13	0.40	-0.04	0.87
Parental Education	0.18	0.25	0.24	0.30
Smoking[3]	-1.16	0.25	-0.07	0.94
In-Scanner Motion	-0.03	0.83	0.09	0.70
Reaction Time	0.13	0.38	0.07	0.78
Cognition	0.11	0.47	-0.12	0.59
Depression	-0.33	0.03	-0.33	0.15
Amotivation	-0.56	<0.001	-0.52	0.016
Anhedonia	-0.40	0.008	-0.30	0.18
Expressivity	-0.40	0.008	-0.29	0.20
Positive Symptoms	-0.32	0.40	-0.27	0.25
Antipsychotic Dose	n/a	n/a	-0.10	0.67

1) r values reported from Pearson's correlations for continuous variables and t values reported for categorical variables; p-values are uncorrected
2) positive t value indicates female > male
3) positive t value indicates smoker > non-smoker

Supplementary Table 2. VS and amotivation relationship to potential confound variables.

The relationships of VS activation and CAINS amotivation are summarized in Supplementary Table 2 above. Across the full sample there were no significant relationships of CAINS amotivation with age, sex, smoking, cognition, motion, or reaction time; but clinical amotivation did relate to education, parental education, depression, CAINS anhedonia, CAINS expressivity, and SAPS global positive symptoms. Within the SZ group, amotivation did not relate

significantly to sex, education, parental education, smoking, depression, antipsychotic dose, cognition, motion, or reaction time; but did relate to age, CAINS anhedonia, CAINS expressivity, and positive symptoms.

VS task activation (contrast parameters extracted from the unbiased bilateral VS ROI) showed the same group and amotivation correlations found in the voxelwise analyses (CT: 6.83 ± 9.24, SZ: 0.46 ± 9.76, group $t=-2.2$, $p=0.03$, Cohen's $d=0.67$; full sample amotivation correlation $r=-0.56$,$p<0.001$; SZ amotivation correlation $r=-0.52$, $p=0.02$). Across the full sample, VS activation did not relate to age, sex, education, parental education, smoking, cognition, motion, or reaction time, but did correlate with depression, anhedonia, expressivity, and positive symptoms. In the SZ group, VS activation did not relate to age, sex, education, parental education, smoking, cognition, motion, reaction time, depression, anhedonia, expressivity, positive symptoms, or antipsychotic dose.

Group Difference in VS Activation, Controlling for Potential Confounds

Confound Variable	Full Sample	
	t*	p
(None)	-2.20	0.032
Age	-1.90	0.061
Sex	-2.20	0.033
Education	-2.03	0.048
Parental Education	-1.95	0.059
Smoking	-2.01	0.051
In-Scanner Motion	-2.18	0.035
Reaction Time	-2.25	0.030
Cognition	-2.14	0.038
Depression	-1.20	0.235
Amotivation	-0.02	0.982
Anhedonia	-1.33	0.190
Expressivity	-0.95	0.348
Positive Symptoms	-0.79	0.435
Antipsychotic Dose	-1.10	0.276

*sign of t-value reflects CT>SZ test, so negative values indicate lower VS in SZ

Amotivation:VS Correlation, Controlling for Potential Confounds

Confound Variable	Full Sample		Within SZ	
	r*	p	r*	p
(None)	-0.56	<0.001	-0.52	0.016
Age	-0.55	<0.001	-0.54	0.031
Sex	-0.56	<0.001	-0.52	0.019
Education	-0.60	<0.001	-0.58	0.011
Parental Education	-0.59	<0.001	-0.49	0.030
Smoking	-0.54	<0.001	-0.52	0.019
In-Scanner Motion	-0.56	<0.001	-0.51	0.021
Reaction Time	-0.59	<0.001	-0.55	0.013
Cognition	-0.56	<0.001	-0.52	0.023
Depression	-0.51	<0.001	-0.47	0.032
Anhedonia	-0.50	0.003	-0.48	0.047
Expressivity	-0.48	0.002	-0.47	0.037
Positive Symptoms	-0.51	0.004	-0.51	0.041
Antipsychotic Dose	-0.54	<0.001	-0.51	0.020

*r values are standardized betas from a regression with z-scored variables, these are equivalent to a Pearson's partial r value

Supplementary Table 3. Group and amotivation effects on VS, controlling for potential confounds

Therefore, we did not identify significant confounds for our key VS findings. Consistent with this, the group difference in VS activation was not substantially changed by including any of these variables, although the significance of the group effect became borderline when including age, smoking, or parental education (see Supplementary Table 3 above). The exception is for psychiatric symptom measures or antipsychotic dosage which when included in the model rendered the group difference non-significant; however, in the control group psychiatric symptoms were minimal and antipsychotic dosage was zero so controlling the group difference for these variables that nearly replicate the categorical distinction is not really meaningful. The full sample amotivation correlation with VS activation was also not substantially altered by including any of these variables (p's all remained <0.01). Similarly, the amotivation correlation with VS activation in the SZ group was not substantially altered by including any of these variables (all p's<0.05). Although we lack the statistical power to detect significant differences between the VS:amotivation correlation compared to the VS correlation with other symptoms that are themselves correlated with amotivation, our results demonstrate that the hypothesized correlation of VS with amotivation is quantitatively stronger and is not driven by other negative symptom domains or by common contributors to secondary negative symptoms such as depression, positive symptoms, or antipsychotic dose, or by cognitive impairment or any other demographic and behavioral variables examined here.

SUPPLEMENTARY REFERENCES

1 First, M. B., Williams, J. B. W., Spitzer R. L., & Gibbon, M. Structured Clinical Interview for DSM-IV-TR Axis I Disorders, Clinical Trials Version (SCID-CT). *New York: Biometrics Research, New York State Psychiatric Institute* (2001).

2 Maxwell, M. E. Family Interview for Genetic Studies (FIGS): Manual for FIGS. *Bethesda, MD: Clinical Neurogenetics Branch, Intramural Research Program, National Institute of Mental Health* (1992).

3 Horan, W. P., Kring, A. M., Gur, R. E., Reise, S. P. & Blanchard, J. J. Development and psychometric validation of the Clinical Assessment Interview for Negative Symptoms (CAINS). *Schizophr Res* **132**, 140-145 (2011).

4 Kirkpatrick, B., Fenton, W. S., Carpenter, W. T., Jr. & Marder, S. R. The NIMH-MATRICS consensus statement on negative symptoms. *Schizophr Bull* **32**, 214-219 (2006).

5 Andreasen, N. C. The Scale for the Assessment of Negative Symptoms (SANS): conceptual and theoretical foundations. *Br J Psychiatry Suppl*, 49-58 (1989).

6 Cannon-Spoor, H. E., Potkin, S. G. & Wyatt, R. J. Measurement of premorbid adjustment in chronic schizophrenia. *Schizophr Bull* **8**, 470-484 (1982).

7 Andreasen, N. C. Scale for the assessment of positive symptoms (SAPS). *University of Iowa, Iowa City.* (1984).

8 Addington, D., Addington, J. & Schissel, B. A depression rating scale for schizophrenics. *Schizophrenia Research* **3**, 247-251 (1990).

9 Heatherton, T. F., Kozlowski, L. T., Frecker, R. C. & Fagerström, K. O. The Fagerström Test for Nicotine Dependence: a revision of the Fagerström Tolerance Questionnaire. *Br J Addict* **86**, 1119-1127 (1991).

10 Hollingshead, A. A. Four-factor index of social status. Unpublished manuscript. *New Haven, CT: Yale University* (1975).

11 Gur, R. C. *et al.* A cognitive neuroscience-based computerized battery for efficient measurement of individual differences: Standardization and initial construct validation. *Journal of Neuroscience Methods* **187**, 254-262 (2010).

12 Pehlivanova, M. *et al.* Diminished cortical thickness Is associated with impulsive choice in adolescence. *J Neurosci* **38**, 2471-2481 (2018).

13 Yu, L. Q. *et al.* Steeper discounting of delayed rewards in schizophrenia but not first-degree relatives. *Psychiatry Research* **252**, 303-309 (2017).

14 Wolf, D. H. *et al.* Amotivation in schizophrenia: integrated assessment with behavioral, clinical, and imaging measures. *Schizophr Bull* **40**, 1328-1337 (2014).

15 Davis, J. M. Comparative doses and costs of antipsychotic medication. *Arch Gen Psychiatry* **33**, 858-861 (1976).

16 Kohler, C. G. *et al.* Facial emotion recognition in schizophrenia: intensity effects and error pattern. *Am J Psychiatry* **160**, 1768-1774 (2003).

17 Bartra, O., McGuire, J. T. & Kable, J. W. The valuation system: a coordinate-based meta-analysis of BOLD fMRI experiments examining neural correlates of subjective value. *Neuroimage* **76**, 412-427 (2013).

18 Senecal, N., Wang, T., Thompson, E. & Kable, J. W. Normative arguments from experts and peers reduce delay discounting. *Judgm Decis Mak* **7**, 568-589 (2012).

19 Kang, M. S. *et al.* Decision value signals in the ventromedial prefrontal cortex and anhedonia across mood and psychotic disorders. Preprint at: *https://www.biorxiv.org/content/10.1101/2020.12.01.407197v1.full* (2020).
20 Miller, E. M., Shankar, M. U., Knutson, B. & McClure, S. M. Dissociating motivation from reward in human striatal activity. *J Cogn Neurosci* **26**, 1075-1084 (2014).
21 Suzuki, S., Lawlor, V. M., Cooper, J. A., Arulpragasam, A. R. & Treadway, M. T. Distinct regions of the striatum underlying effort, movement initiation and effort discounting. *Nat Hum Behav*, **5**, 378-388 (2020).
22 Strauss, G. P. *et al.* Avolition in schizophrenia is associated with reduced willingness to expend effort for reward on a Progressive Ratio task. *Schizophrenia research* **170**, 198-204 (2016).
23 Reddy, L. F. *et al.* Effort-based decision-making paradigms for clinical trials in schizophrenia: part 1—psychometric characteristics of 5 paradigms. *Schizophr Bull* **41**, 1045-1054 (2015).
24 Bialaszek, W., Marcowski, P. & Ostaszewski, P. Physical and cognitive effort discounting across different reward magnitudes: Tests of discounting models. *PLoS One* **12**, e0182353 (2017).
25 Le Bouc, R. *et al.* Computational dissection of dopamine motor and motivational functions in humans. *J Neurosci* **36**, 6623-6633 (2016).
26 Cooper, J. A. *et al.* Effortful goal-directed behavior in schizophrenia: Computational subtypes and associations with cognition. *J Abnorm Psychol* **128**, 710-722 (2019).
27 Prévost, C., Pessiglione, M., Météreau, E., Cléry-Melin, M. L. & Dreher, J. C. Separate valuation subsystems for delay and effort decision costs. *J Neurosci* **30**, 14080-14090 (2010).
28 Hartmann, M. N. *et al.* Apathy but not diminished expression in schizophrenia is associated with discounting of monetary rewards by physical effort. *Schizophr Bull* **41**, 503-512 (2015).
29 Seaman, K. L. *et al.* Subjective value representations during effort, probability and time discounting across adulthood. *Soc Cogn Affect Neurosci* **13**, 449-459 (2018).
30 Chong, T. T. *et al.* Computational modelling reveals distinct patterns of cognitive and physical motivation in elite athletes. *Sci Rep* **8**, 11888 (2018).
31 Mitchell, S. H. Effects of short-term nicotine deprivation on decision-making: delay, uncertainty and effort discounting. *Nicotine Tob Res* **6**, 819-828 (2004).
32 Culbreth, A. J., Moran, E. K. & Barch, D. M. Effort-cost decision-making in psychosis and depression: could a similar behavioral deficit arise from disparate psychological and neural mechanisms? (2018).
33 Ehrlich, S. *et al.* Striatal function in relation to negative symptoms in schizophrenia. *Psychological Medicine* **42**, 267-282 (2012).
34 Mucci, A. *et al.* Is avolition in schizophrenia associated with a deficit of dorsal caudate activity? A functional magnetic resonance imaging study during reward anticipation and feedback. *Psychological medicine* **45**, 1765-1778 (2015).
35 Stepien, M. *et al.* Investigating the association of ventral and dorsal striatal dysfunction during reward anticipation with negative symptoms in patients with schizophrenia and healthy individuals. *PLoS One* **13**, e0198215 (2018).
